# Mandibular biomechanics rehabilitated with different prosthetic restorations under normal and impact loading scenarios

**DOI:** 10.1186/s12903-024-04681-0

**Published:** 2024-08-15

**Authors:** Yomna H. Shash

**Affiliations:** Biomedical Engineering Department, Faculty of Engineering, Helwan University, Cairo, Egypt

**Keywords:** Fixed restorations, Zirconia, BIOHPP, Mandibular movement, Fractures

## Abstract

**Background:**

Restorative treatment options for edentulous patients range from traditional dentures to fixed restorations. The proper selection of materials greatly influences the longevity and stability of fixed restorations. Most prosthetic parts are frequently fabricated from titanium. Ceramics (e.g. zirconia) and polymers (e.g. PEEK and BIOHPP) have recently been included in these fabrications. The mandibular movement produces complex patterns of stress and strain. Mandibular fractures may result from these stresses and strains exceeding the critical limits because of the impact force from falls or accidents. Therefore, it is necessary to evaluate the biomechanical behavior of the edentulous mandible with different restorations under different loading situations.

**Objective:**

This study analyzes the biomechanical behavior of mandibles after four prosthetic restorations for rehabilitation under normal and impact loading scenarios.

**Material and Methods:**

The mandibular model was constructed with a fixed restoration, which was simulated using various materials (e.g. Titanium, Zirconia & BIOHPP), under frontal bite force, maximum intercuspation, and chin impact force. From the extraction of tensile and compressive stresses and strains, as well as the total deformation of mandible segments, the biomechanical behavior and clinical situations were studied.

**Results:**

Under frontal bite, the anterior body exhibited the highest tensile (60.34 MPa) and compressive (108.81 MPa) stresses using restoration 4, while the condyles and angles had the lowest tensile (7.12 MPa) and compressive (12.67 MPa) stresses using restoration 3. Under maximum intercuspation, the highest tensile (40.02 MPa) and compressive (98.87 MPa) stresses were generated on the anterior body of the cortical bone using restoration 4. Additionally, the lowest tensile (7.7 MPa) and compressive (10.08 MPa) stresses were generated on the condyles and angles, respectively, using restoration 3. Under chin impact, the highest tensile (374.57 MPa) and compressive (387.3 MPa) stresses were generated on the anterior body using restoration 4. Additionally, the lowest tensile (0.65 MPa) and compressive (0.57 MPa) stresses were generated on the coronoid processes using restoration 3. For all loading scenarios, the anterior body of the mandible had the highest stress and strain values compared with the other segments. Compared to the traditional titanium restoration.2, restoration.1(zirconia) increases the tensile and compressive stresses and strains on the mandibular segments, in contrast to restoration.3 (BIOHPP). In addition, zirconia implants exhibited higher displacements than the other implants.

**Conclusion:**

In the normal loading scenario, the tensile and compressive stresses and strains on the mandible were within the allowable limits when all restorations were used. Under the chin impact loading scenario, the anterior body of the mandible was damaged by restorations 1 and 4.

## Introduction

The mandible is the largest, strongest, and lowest bone in the human face, holding the lower teeth firmly in place. There are various segments of the mandible, including the anterior body, angle, ramus, condyle, and coronoid processes. The rounded end of the bone that slides into the moveable junction between the bone and skull is called the condyle, whereas the coronoid process is the triangle protrusion of the mandible. The ascending ramus is the straighter, flatter portion of the jaw that connects the coronoid process and condyles to the mandible's body. The junction of the mandibular body's inferior border and the ascending ramus's posterior edge produce the mandibular angle (gonial angle). The two joints that attach the mandible to the skull are temporomandibular joints (TMJ). These synovial joints permit the intricate three-dimensional movements required for life. Mandibular depression, elevation, lateral deviation (which happens on both the left and right sides), retrusion, and protrusion are the five movements of the TMJ [1, 2].

The mandible is the only bone in the skull that can move and rests behind the maxilla. The mandibular movement is a complicated series of connected three-dimensional rotational and translational activities [2]. Hence, each segment in the mandible develops a complex pattern of tensile, compressive, shear, and torsional stresses and strains. These stresses and strains may exceed the critical limits because of the impact forces from falls, accidents, and other situations, causing mandibular fractures [3]. Hence, it is necessary to evaluate the biomechanical behavior of the mandible in each loading situation.

The loss of natural teeth is referred to as “edentulism” [4]. It is not just a problem for the elderly; many young individuals also experience it for several reasons. Edentulism raises the risk of several diseases, induces bone resorption, reduces the ability to chew, impairs speech, and leads to an unattractive appearance [5]. Additionally, in most cases of edentulous patients, degenerative changes in the TMJ may appear with age [6]. Therefore, the use of kinematic face bows is mandatory in this situation to restore the proper function of the joint [7]. As a novel technology in restorative dentistry, the MODJAW is used to integrate the dynamics within morphological data. The MODJAW is a high-frequency camera that quickly analyzes patient-specific functional motions and animates static 3D models. With the aid of this advanced technology, TMJ issues can be diagnosed precisely, and the movements of the condyles during jaw movements can be visualized precisely [8].

For edentulous patients, to restore quality of life and eliminate the issues of edentulism, “complete dentures”, “implant removable dentures” and “implant-supported fixed restorations” are used as acceptable treatment options [9]. Complete dentures are less expensive than other restorations and are simple to use and replace [10]. However, the design of complete dentures involves many factors, none of which should be disregarded, as they may result in a failed denture and cause complications after placement [11]. The most frequent complaints of patients wearing complete dentures are mucosal irritation, inadequate retention and stability, food accumulation under the dentures, speech difficulties, inefficient chewing, unsightly appearance, broken dentures, and deboning of teeth [11].

The existing drawbacks of conventional complete dentures have prompted scientific and technological advances to focus on developing innovative remedies. When the All-on-4 treatment approach is combined with the most recent developments in dental implants, treatment times, morbidity rates, and other potential risks are typically decreased in edentulous patients. This approach has been used more regularly and has become more common because it has developed specifically to address the complex prosthetic and surgical issues brought on by anatomical restrictions [12]. In addition, fixed restorations preserve bone quality and quantity while enhancing mastication, speech, and patient self-esteem by minimal surgical intervention [13].

In fixed prosthetic restorations (hybrid restorations), four implants are positioned in the jaw body and joined by a superstructure framework. The artificial teeth are positioned and fastened to the framework using an acrylic material. Polymethyl methacrylate (PMMA) and polyamide-12 are commonly used in dentistry as denture bases because of their good properties and ease of application [13]. However, the esthetic appearance of removable dentures with PMMA bases can be impaired by the visibility of metal clasps. Polyamide-12 is a viable substitute for PMMA-based dentures [14]. Polyamide 12’s flexibility enables retentive materials to be matched to the color of teeth and gums. Although the polyamide-12 denture material achieves good fracture resistance, its elastic modulus is too low to match that of the PMMA materials. Hence, polyamide-12 needs to be modified to regularly yield superior quality to PMMA materials [15].

One of the key factors for long-term clinical success is appropriate planning of the materials used for the implant prosthesis’s substructure and superstructure. The properties of the material and spatial geometric configuration model of each part significantly influence the transmission of functional loads and stress distribution in a bone-implant-prosthesis assembly [13]. Titanium has been the traditional material used for the production of most prosthetic parts, including frameworks, screws, and implant systems. Titanium has many benefits, making it a popular material for dental work with excellent success rates [16]. It is a flexible and useful biomaterial because of its physiological inertia, biocompatibility, resistance to corrosion in oral settings, and combination of strength and lightness. As a result, its significance in the dentistry market has increased. Nevertheless, despite all of its benefits and reputation for affordability and quick availability, the technologies associated with casting, welding, and machining remain costly and have significant drawbacks. Therefore, the wide use of titanium in dental prostheses depends on technological advances and more laboratory and clinical investigations to develop more profitable techniques that prove the efficiency of titanium [16]. In addition, titanium material can induce hypersensitive reactions such as erythema, urticaria, eczema, swelling, pain, and necrosis [17].

Ceramics and polymers have recently been used in fixed restorations as alternatives to titanium [18, 19]. One viable alternative to titanium is the zirconia bioceramic(Y-TZP), due to its rigidity, durability, good mechanical properties (strong in compression), and good chemical properties [20, 21]. Owing to its biocompatibility and excellent mechanical properties, zirconia has been successfully used in recent years as a dental biomaterial. This rigid material has been used in the fabrication of implants, frameworks, crowns, screws, and teeth because it is expected to protect the bone, preventing bone resorption, according to Carames et al*.* [22]. Zirconia satisfies both the combined need for exceptional strength and excellent esthetics, unlike previous types of all-ceramic restorations. It also satisfies the need for people with metal allergies to undergo an all-ceramic restoration [23]. Zirconia implants, abutments, and crowns provide high flexural strength, good esthetics and exhibit good tissue compatibility, and low plaque accumulation. Patients with bruxism can benefit from zirconia-based teeth because polished zirconia lessens antagonistic tooth wear more than other materials. The dentist can create well-fitting restorations with precision and little chair side adjustment using computer-aided design and manufacturing [20, 22, 23].

In contrast to rigid materials, some researchers have hypothesized that soft polymers can reduce occlusal forces and evenly distribute loads, thereby reducing bone resorption and restoration failure [24, 25]. BIOHPP (Bredent Medical, Weissenhorner, Germany [26]) is an innovative high-performance polymer that has recently been used in dentistry for implant-supported fixed restorations. It contains 20% ceramic filler particles scattered throughout the PEEK matrix, with grain sizes ranging from 0.3 to 0.5 µm. The exceptional mechanical properties are attributed to the consistent homogeneity, which is achieved by the extremely fine grain sizes of the ceramic particles [27, 28]. Furthermore, BIOHPP is distinguished by its biocompatibility, meaning that it does not interact with other materials, is suitable for people with allergies, and has high resistance to gamma and X-rays (not subject to the formation of artifacts). It is durable and thus the antagonist’s teeth are not subject to abrasion. In addition, because of its flexibility, low weight, and ability to absorb shock, BIOHPP may produce fewer stresses on bone and other components and distribute the chewing force uniformly without overloading when used in fixed restorations [28, 29].

Despite the recent usage of zirconia and BIOHPP in dentistry, further study is still needed to completely understand the implications of using these materials as the primary components of fixed prosthetic restorations and their effects on the biomechanics of the edentulous mandible. In the field of dentistry, the finite element method (FEM) provides an alternative solution to the physical model due to its ability to accurately represent complex geometries, modify models, suggest alternative designs, stimulate various materials under various conditions, and extract the internal stresses and strains in any part involved in restoration [30, 31]. In addition, previous studies have demonstrated that FE analysis is a reliable method for evaluating the biomechanical stability of mandibles that have been rehabilitated using various plates, implanting systems, and osteosynthesis materials [32, 33]. The objective of this study is to use the finite element method to evaluate the biomechanical behavior of mandibles that are rehabilitated with four fixed prosthetic restorations to assess their responses under normal and impact loading scenarios. The fixed prosthetic restorations are:

**Table:** 

Restoration.1	Zirconia framework and implants, with acrylic teeth
Restoration.2 (Traditional)	Titanium framework and implants with acrylic teeth
Restoration.3	BIOHPP framework and implants with acrylic teeth
Restoration.4	Zirconia framework and BIOHPP implants with zirconia teeth

In this study, the mandible is divided into five segments: the anterior body (with inner volume of trabecular bone), ramus, angle, coronoid process, and condyle. For all segments, the biomechanical behavior and the clinical situations are analyzed and discussed from the extraction of the tensile and compressive stresses and strains and the total deformations. In addition, the total displacements of prosthetic implants with different materials are calculated to evaluate the stability of fixed prosthetic restorations under different loading scenarios.

## Materials and methods

### Model generation

#### Mandibular model

The most commonly used method for creating 3D anatomical models of the mandible is computed tomography (CT), which offers details on tissue density and contrast enhancement. First, image acquisition may be tailored to ensure that the appropriate anatomic structures are well visualized, which can greatly simplify the process of creating patient-specific models with high-quality content. The following parameters are relevant for any image acquisition: volumetric data acquisition, minimal image artifacts, high signal-to-noise ratios, contrast-to-noise ratios, small slice thicknesses, and high spatial resolution. Next, image segmentation is crucial for generating accurate patient-specific 3D anatomical models. Partitioning a volumetric medical image into distinct regions—typically organs or sick structures—is the aim of image segmentation. After image segmentation, the 3D surface mesh is exported into STL format. Subsequently, the STL files are converted to OBJ format for export to a program (like Solidworks, Spaceclaim, etc.) to create the required design [34].

The edentulous mandible’s 3D geometry, measuring 120 mm in length, 60 mm in height, and 30 mm in symphysis height, was obtained as an OBJ file from the “BodyParts3D/Anatomography” website (BodyParts3D, Life Sciences Integrated Database Center, Japan [35]). In “Space Claim” program, the mandibular model was converted to a solid, modified, and repaired. In the repair process, the initial step was to fix, reduce, and smooth the facets. After solidification, a second repair process was performed to minimize intricate faces and curves, combine faces, eliminate small faces, and correct poor faces, missing faces, curves, and gaps.

**Fig. 1:**
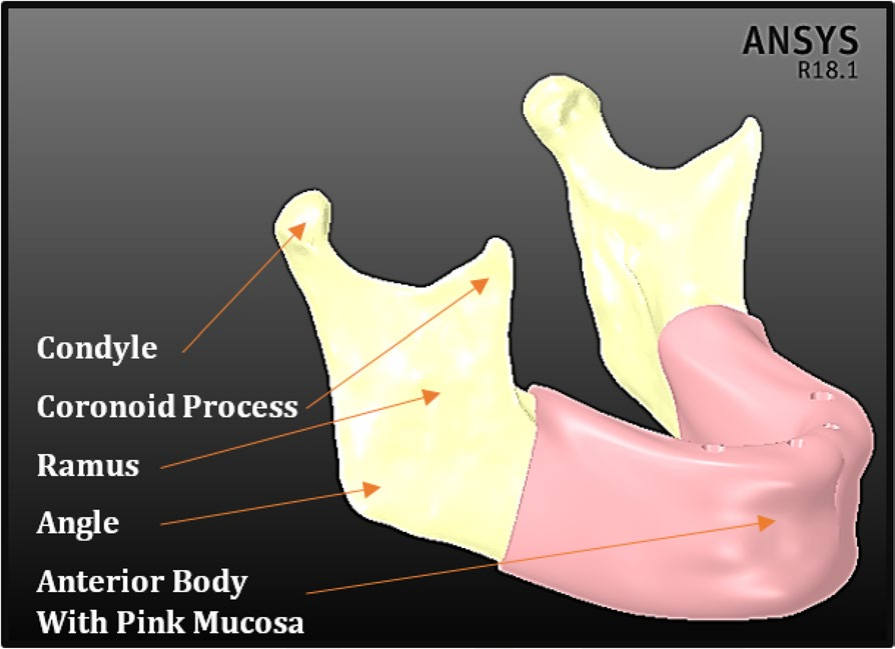
Mandibular model with mucosa

The anterior body of the mandible was then divided into cortical bone with a thickness of 2 mm and inner volume from the trabecular bone. The posterior part of the mandible was divided into four segments: the ramus, angle, coronoid process, and condyle. In addition, the mandible is covered with a 2-mm thick mucosa, as shown in Fig. 1.

#### Fixed prosthetic restoration

##### Implants

**Fig. 2:**
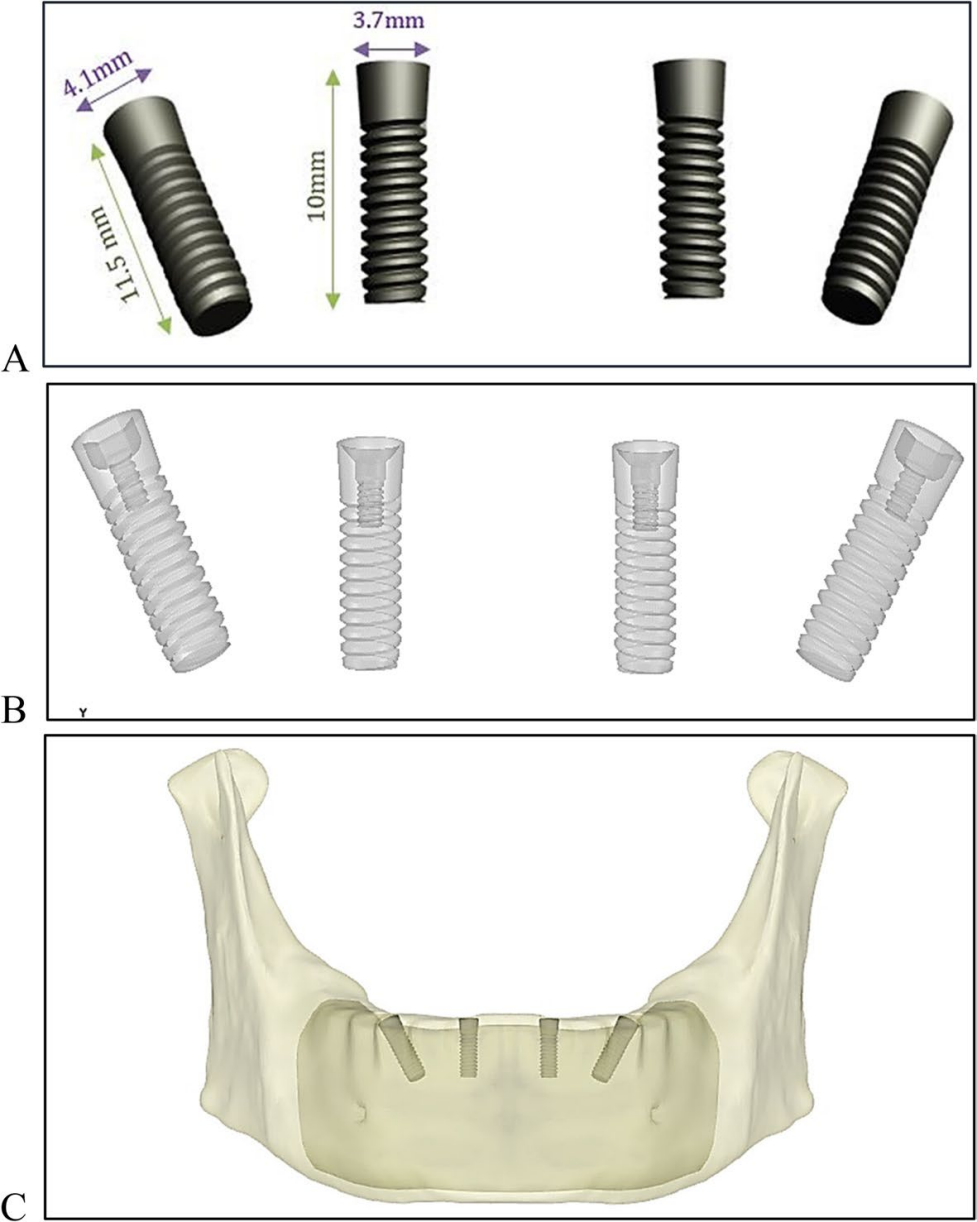
Implant design: (**A**) Dimensions of anterior and posterior implants, (**B**) transparent view, and (**C**) implants placed on the bone margin

In the first stage, to construct the infrastructure of the fixed restoration, four threaded implants were modeled and placed across the anterior body of the mandible (Fig. 2). The implants were constructed in “Space Claim” program with the dimensions illustrated in the ZIMMER catalog (ZIMMER Dental, Tapered Screw-Vent®Implant System, Biomet Dental, USA [36]), as shown in Figs. 2A and B. Two posterior implants measuring 4.1 mm in diameter and 11.5 mm in height, angled at 30°, were placed in the second premolar region, and two anterior implants measuring 3.7 mm in diameter and 10 mm in length were placed in the lateral incisor region. The platform of each implant was measured to be 3.5 mm. Each implant showed complete osteointegration and was placed on the bone margin, as shown in Fig. 2C.

##### Multi-unit abutments

**Fig. 3:**
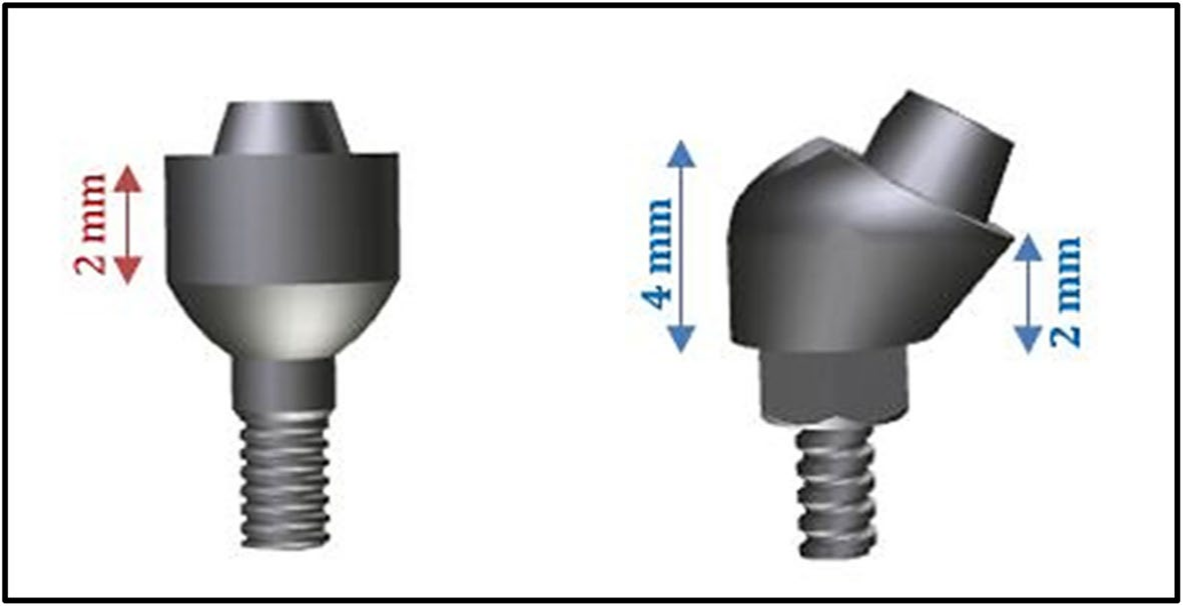
Anterior and posterior abutments

In the “Space Claim” program, the anterior implants with platform diameters of 3.5 mm were fitted with straight tapered abutments with cuff heights of 2 mm (Fig. 3). These tapered abutments do not engage the internal hex connection (ZIMMER Dental, Tapered Screw-Vent®Implant System, Biomet Dental, USA [36]). For the posterior implants, angled abutments with cuff heights of 2 mm / 4 mm were used (Fig. 3). Straight and angled tapered abutments have been used for multiple-unit, screw-retained restorations.

**Fig. 4:**
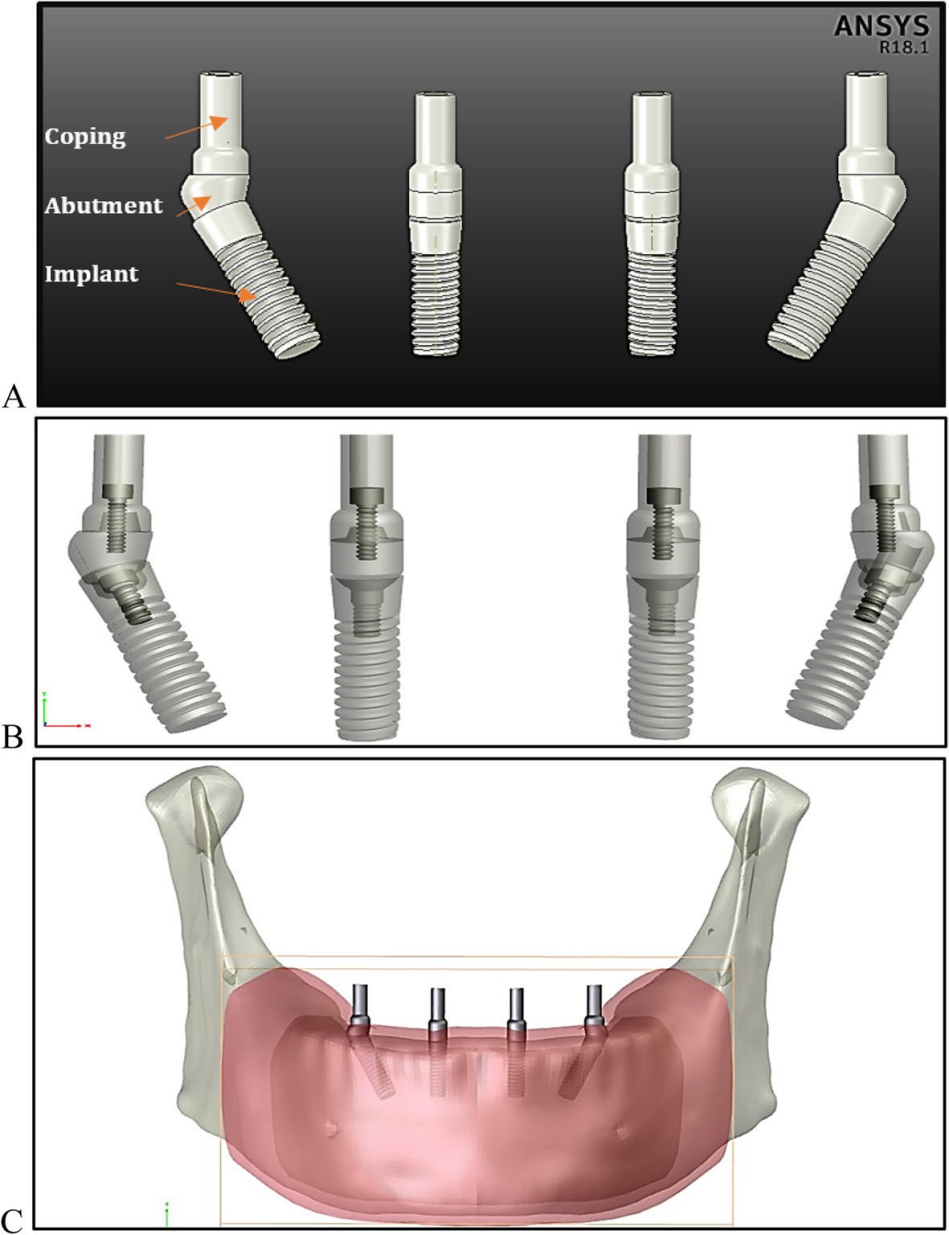
Reconstruction of the infrastructure of the fixed prosthetic restoration: **A**) Implants, abutments, copings, and screws; **B**) transparent view and **C**) infrastructure of the fixed restoration mounted on mandible

Four sleeves or copings were fitted into the straight and angled abutments to support the superstructure in the next stage, as shown in Fig. 4.

##### Framework and artificial teeth

**Fig. 5:**
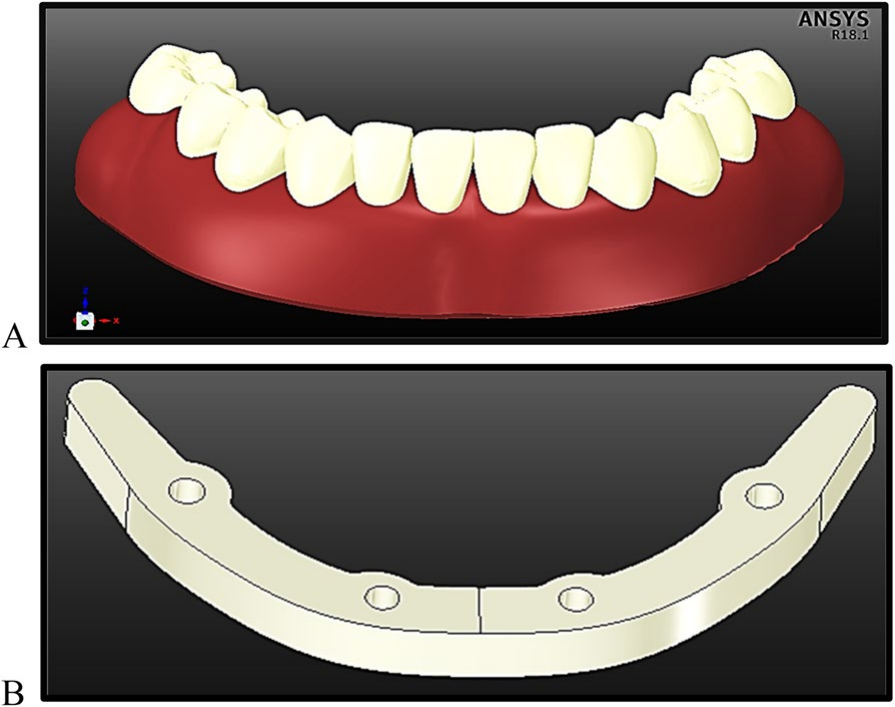
The superstructure of the fixed prosthetic restoration: **A**) the denture base and artificial teeth, and **B**) the framework

In the second stage, the superstructure (Fig. 5) was constructed. The framework was fastened to the copings over the abutments and secured with acrylic material to support the artificial teeth. This framework provides a secure support for the artificial teeth, minimises the number of required implants and improves load distribution [37]. The framework dimensions were 5 mm for width, 5.5 mm for thickness, and 10 mm for distal extension. It was shaped like a horseshoe to fit the curvature of the mandible. Twelve prosthetic teeth were positioned and cemented to the framework using acrylic material. Implant-supported fixed restorations frequently have fewer posterior teeth than removable dentures, because their cantilever extensions are limited to the first molars [13]. In addition, the fixed prosthetic restorations are frequently 15 mm from the mucosal surface.

**Fig. 6:**
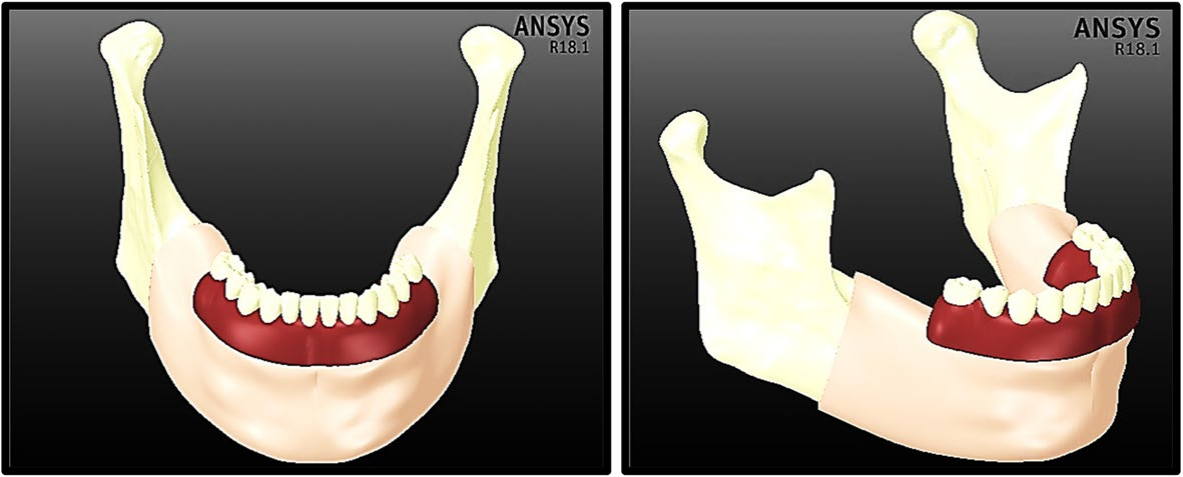
Final model, in “Space Claim” program

Finally, the superstructure was mounted on the infrastructure using mini screws with diameters of 1.25 mm. The final model is illustrated in Fig. 6.

### Mesh generation

**Fig. 7:**
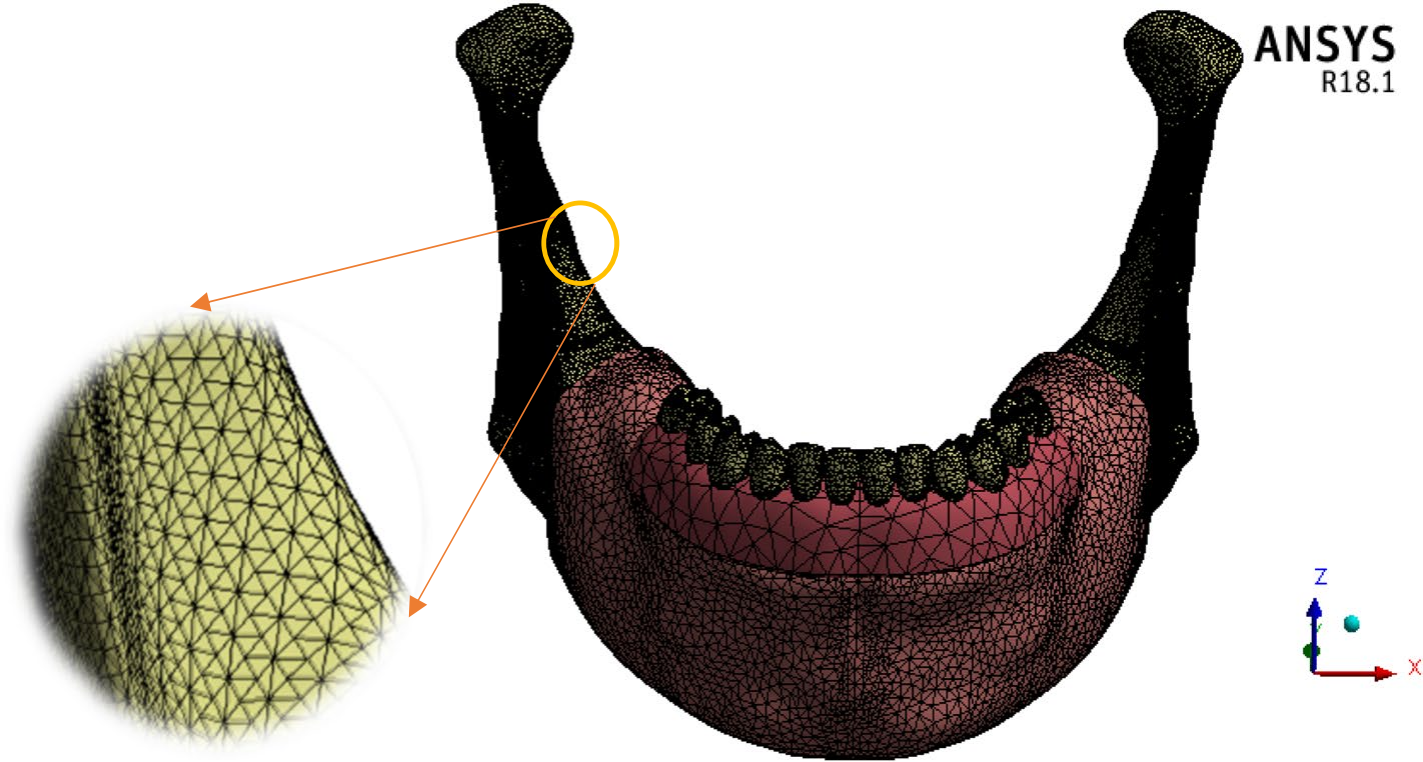
Meshing

**Fig. 8:**
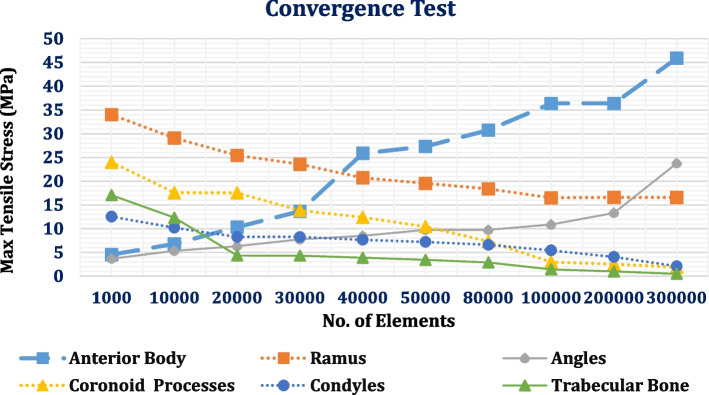
Convergence study: effect of increasing the number of elements on the maximum tensile stresses of cortical and trabecular bones under frontal bite loading

The “Adaptive” size function with 0.4–2.0 mm element size was used in the ANSYS program, resulting in a large mesh of 584,526 elements and 715,910 nodes (Fig. 7). The mesh refinement was established based on the convergence test. Figure 8 illustrates the influence of increasing the number of elements on the maximum tensile stresses of the cortical and trabecular bones under frontal bite loading. The numbers of nodes and elements in each part are detailed in Table 1. To maintain uniformity throughout the finite element investigations, the same mesh was used for all loading scenarios.

**Table 1:** Number of nodes and elements in each part

	**No. of Elements**	**No. of Nodes**
Mucosa	102,366	18,048
Cortical Bone		
•Anterior Body	117,210	210,530
•Ramus	127,286	73,768
•Angles	64,699	111,210
•Coronoid Processes	18,965	34,322
•Condyles	23,194	40,178
Trabecular Bone	29,283	52,049
Denture Base and Teeth	32,058	57,103
Framework	1679	3241
Copings	3990	7348
Screws	8970	15,906
Abutments	12,521	22,198
Implants	42,305	70,009

### Material properties

The edentulous mandible is composed of trabecular and cortical bone. The mandibular volume consists of five symmetric segments, the anterior body, angle, ramus, condyle, and coronoid processes. Each segment is treated as an orthotropic material because is more accurate than the isotropic approximation for studying the mandibular biomechanics. Table 2 illustrates the orthotropic properties of the cortical and trabecular bones, as reported by Caraveo et al*.* research [33].

**Table 2:** Orthotropic properties of the mandible [33]

		**Anterior Body**	**Angle**	**Ramus**	**Condyle**	**Coronoid Process**	**Trabecular Bone**	**REF**
**Elastic Modulus** **(MPa)**	**Ex**	12,700	12,757	12,971	12,650	14,000	7930	[33]
	**Ey**	21,728	23,793	24,607	23,500	28,000	7930	
	**Ez**	17,828	19,014	18,357	17,850	17,500	7930	
**Poison Ratio**	**Vxy**	0.45	0.41	0.38	0.32	0.28	0.3	
	**Vyx**	0.20	0.22	0.23	0.25	0.28	0.3	
	**Vxz**	0.34	0.30	0.28	0.24	0.23	0.3	
**Shear Modulus (MPa)**	**Gxy**	5,533	5,493	5,386	5,500	5,750	3,050	
	**Gyx**	5,083	4,986	5,014	5,150	5,300	3,050	
	**Gxz**	7,450	7,579	7,407	7,150	7,150	3,050	

In this study, four fixed prosthetic restorations were used to investigate their effects on the mandibular biomechanics. These restorations were restoration.1 (zirconia framework and implants, with acrylic teeth), restoration.2 (titanium framework and implants, with acrylic teeth), restoration.3 (BIOHPP framework and implants, with acrylic teeth), and restoration.4 (zirconia framework and BIOHPP implants, with zirconia teeth). Table 3 presents the isotropic properties of the mucosa and the materials used in fixed prosthetic restorations [38].

**Table 3:** Isotropic properties of the mucosa and different materials used for fixed restorations

	**Elastic Modulus (MPa)**	**Poison Ratio**	**REF**
Mucosa	1	0.40	
Titanium (Ti–6A1–4 V)	110,000	0.33	[38]
Zirconia (Y-TZP)	210,000	0.23	
BIOHPP	4,000	0.40	

### Loading scenarios

#### Normal frontal bite

**Fig. 9:**
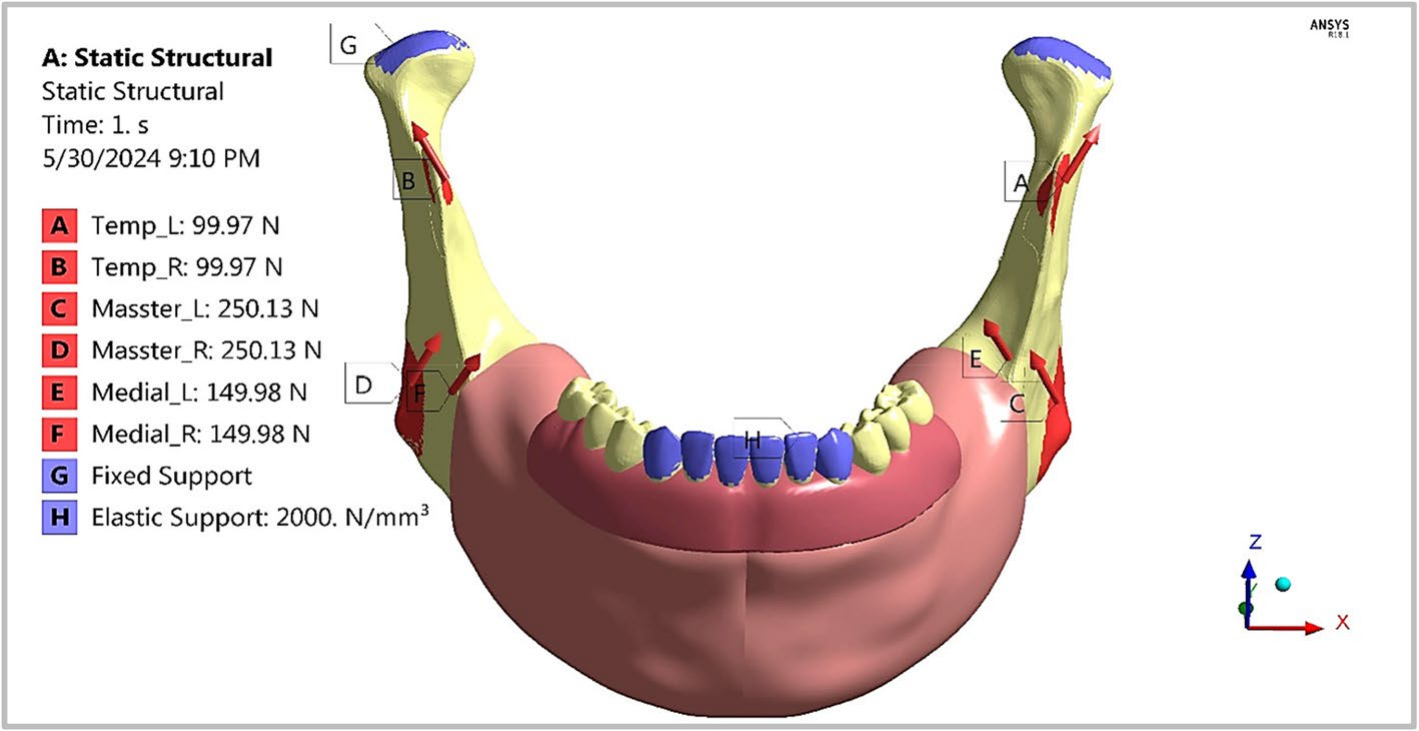
Frontal bite loading scenario

In this section, to stimulate the normal loading scenario of a frontal bite, a stiff body was pressed by the six frontal artificial teeth (two canines, two central incisors, and two lateral incisors), which were represented by elastic support with a stiffness of 2000 N/mm3 in this region [32]. To imitate the normal loading conditions of the mandible, the forces produced by the three main muscles involved in the mastication process—the temporal, masseter, and medial pterygoid—were applied on the mandible, which was rehabilitated with different prosthetic restorations (Fig. 9  and Table 4). The surfaces on which each of the six muscles acted were determined approximately from the information gleaned from the anatomy and physiology of the stomatognathic system. The values and directions of the muscle forces were estimated by Gregolin et al*.* [39].

**Table 4:** Values and directions of the mastication forces of the six muscles

Muscle force (*N*)	X	Y	Z
Left Temporalis	50	22.1	83.7
Right Temporalis	-50	22.1	83.7
Left Masseter	-104.7	-51.7	221.2
Right Masseter	104.7	-51.7	221.2
Left pterygoid	-55.8	-72.9	118.6
Right pterygoid	55.8	-72.9	118.6

#### Maximum intercuspation

**Fig. 10:**
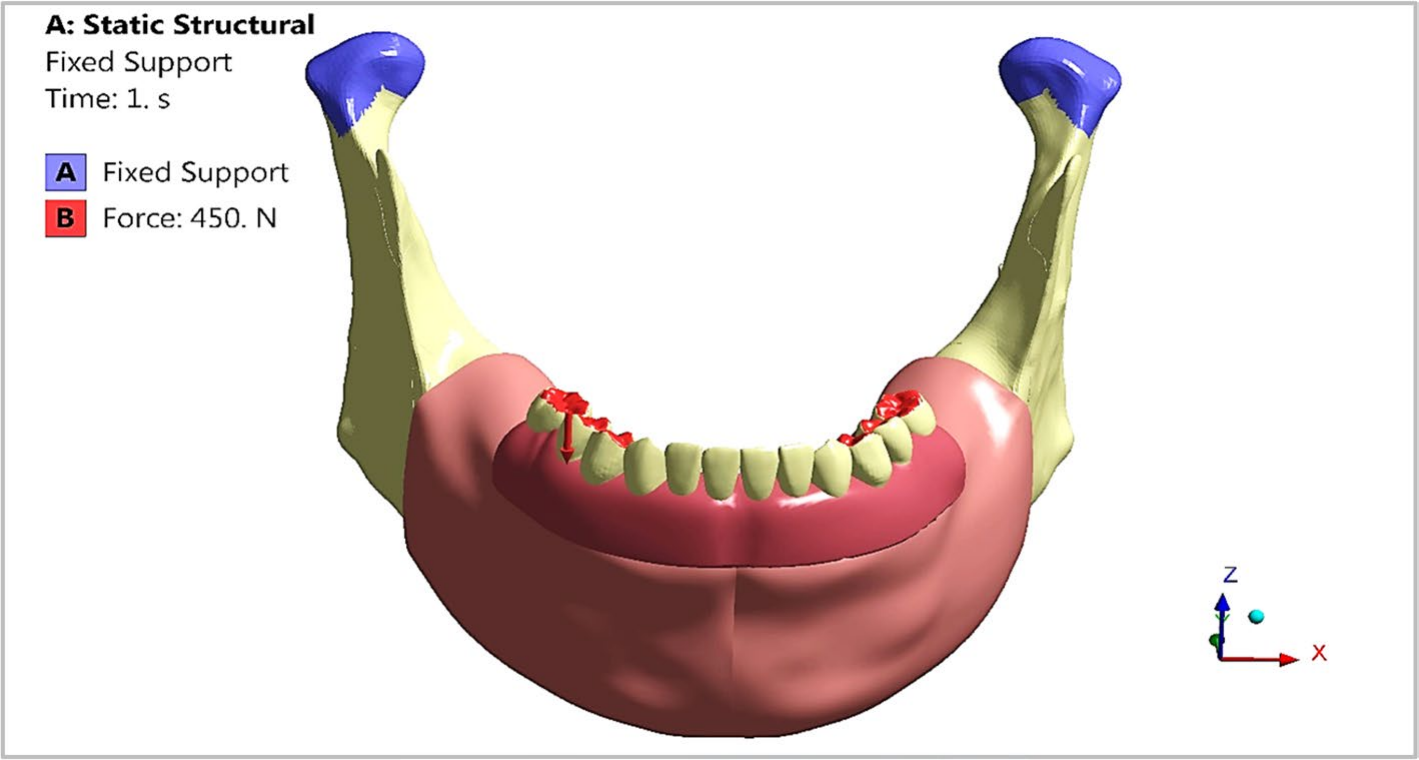
Maximum intercuspation loading scenario

In maximum intercuspation, the opposing teeth are in complete intercuspation or maximum meshing independently of the condylar position. To stimulate posterior maximum intercuspation during bilateral mastication, the posterior artificial teeth (first premolar, second premolar, and first molar) of the fixed restoration in the mandible were nearly in complete contact with the posterior teeth in the maxilla, as presented in Fig. 10. An average force of 450 N was distributed on the posterior teeth to stimulate normal conditions according to [40], while the condyles were in fixed support.

#### Chin impact

**Fig. 11:**
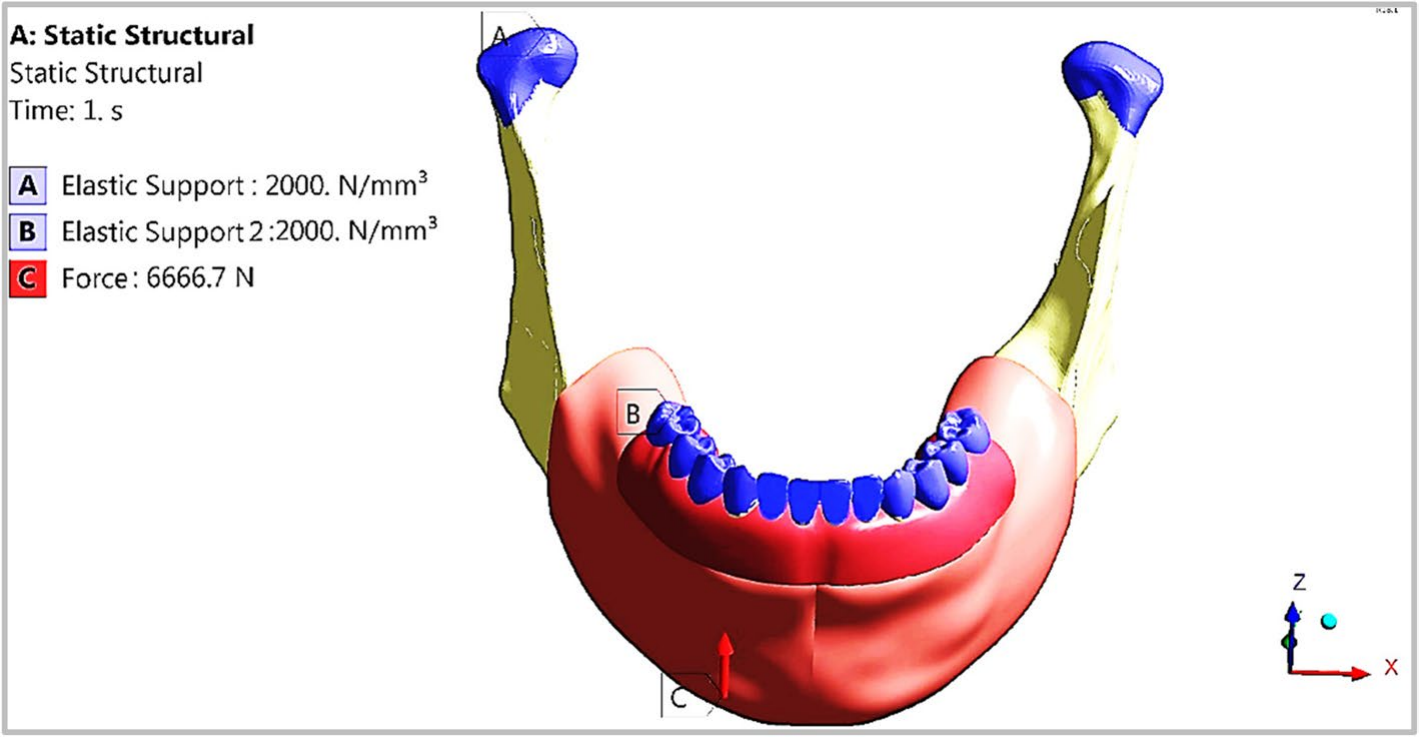
Chin impact loading scenario

In traumatizing circumstances, the most vulnerable jaw impact locations are the chin (in the case of a fall or a frontal hit) and the lateral portion of the jaw (in the case of a fall or a side impact). This part simulated the mandibular exposure to an impact force in the chin area (due to a frontal strike or fall), as shown in Fig. 11. In this loading scenario, the mouth was closed, and the masticatory muscles were relaxed. As a result, the impact was the only force applied to the mandible. The temporomandibular joints were fixed, and the forces were applied in the chin area. The impact force was applied perpendicular to an area of 10 3 m^2^ in the chin. As per [32], the force that resulted from a five-kilogram body (the head) falling from a height of two meters is given by (*F* = 6666.7 N). Upon the chin impact, the mandibular artificial teeth were restricted in mobility by the maxillary teeth. Therefore, artificial teeth had an elastic support with a stiffness of 2000 N/mm3, as the stiffness of the teeth.

### Results evaluation

In the ANSYS program, the maximum displacements and peak maximum (tensile) and minimum (compressive) principal stresses and strains were computed for mandibular segments with orthotropic properties to be used in the evaluation step for investigating the mandibular biomechanics.

In Hooke’s law [41, 42], the nine elastic constants in orthotropic constitutive equations comprise three Young moduli (Ex, Ey & Ez), three Poisson ratios (Vxy, Vyz &Vzx), and three shear moduli (Gxy, Gyz &Gzx). The compliance matrix takes the following form, where symbol (σ) is the stress and $\left(\mathrm\varepsilon\right)$ is the strain produced in different directions:

$$\begin{bmatrix}\mathrm\varepsilon xx\\\mathrm\varepsilon yy\\\mathrm\varepsilon zz\\\mathrm\varepsilon yz\\\mathrm\varepsilon zx\\\mathrm\varepsilon xy\end{bmatrix}=\begin{bmatrix}\frac1{Ex}-\frac{Vyx}{Ey}-\frac{Vzx}{Ez}000\\-\frac{Vxy}{Ex}\frac1{Ey}-\frac{Vzy}{Ez}000\\-\frac{Vxz}{Ex}-\frac{Vyz}{Ey}\frac1{Ez}000\\000\frac1{2Gyz}00\\0000\frac1{2Gzx}0\\00000\frac1{2Gxy}\\\end{bmatrix}\begin{bmatrix}\sigma xx\\\sigma yy\\\sigma zz\\\sigma yz\\\sigma zx\\\sigma xy\end{bmatrix}$$

When direct stresses are applied to a body, then there exists a plane of the body in which the shear stress values are zero. This plane is called the principal plane, and hence, the normal stresses and strains in this plane are called the principal stresses and strains. The three principal stresses are usually labeled as ${\upsigma }_{1},{\upsigma }_{2} \& {\upsigma }_{3}$. ${\upsigma }_{1}$ is the maximum principal stress (most tensile stress), ${\upsigma }_{3}$ is the minimum principal stress (most compressive stress), and ${\upsigma }_{2}$ is the intermediate principal stress. The three principal strains ${\upvarepsilon }_{1},{\upvarepsilon }_{2} \& {\upvarepsilon }_{3}$ can be calculated from the principal stresses using Hook’s law [41, 43].

For the mandibular segments, after extracting the peak maximum principal stresses (tensile stresses) and peak minimum principal stresses (compressive stresses), the values were compared to the limits based on the principal stress theory (Rankine Theory). This theory states that failure occurs when the peak maximum principal stress $({\upsigma }_{1})$ in the complex system reaches the value of the yield limit in the tension test $({\upsigma }_{\text{YT}})$, or the peak minimum principal stress ${(\upsigma }_{3})$ reaches the value of the yield limit in compression test ${(\upsigma }_{\text{YC}})$ [44].

$$\begin{array}{ccc}\text{Material fails if}& {\upsigma }_{1} \ge {\upsigma }_{\text{YT}}& \text{In tension}\\ & {|\upsigma }_{3}|\ge {\upsigma }_{\text{YC}}& \text{In compression}\end{array}$$

The maximum strain criterion (e.g., Tsai and Hahn (1980), Daniel and Ishai (1994), and Herakovich (1998)) is identical to the maximum stress criterion, but its limits are expressed in terms of the strain components [45].The peak maximum principal strains (tensile strains) and peak minimum principal strains (compressive strains) were compared to the allowable limits because of the potential for microdamage and bone resorption due to the concentration and distribution of excessive strains, according to the principal strain theory (Saint–Venant criterion).This theory states that material failure occurs when the peak maximum principal strain $\left(\mathrm\varepsilon1\right)$ reaches the strain corresponding to the yield point under tension test $\left(\mathrm\varepsilon{}_\text{YT}{}\right)$, or when the peak minimum principal strain $\left(\mathrm\varepsilon3\right)$ reaches the strain corresponding to the yield point under compression test $(\mathrm\varepsilon{}_\text{YC})$. This theory is more appropriate for ductile, brittle, and biological materials [44, 46, 47].

$$\begin{array}{ccc}\text{Material fails if}&\mathrm\varepsilon{}_1\geq{\mathrm\varepsilon}_{\mathrm{YT}}&\text{In tension}\\&{\vert{\mathrm\varepsilon}_3}\vert\geq{\mathrm\varepsilon}_{\mathrm{YC}}&\text{In compression}\end{array}$$

The limits for human cortical bone (5 segments) have been determined to be approximately 100–170 MPa for the tensile yield strength and 140–200 MPa for the compressive yield strength according to [38], and other research has determined the value to be approximately 180 MPa in both tension and compression [32]. The limits for the trabecular bone are 12–20 MPa values for the tensile yield strength and 16–30 MPa for compressive yield strength [38]. In addition, microstrain levels of 5000 have been typically indexed as the failure threshold for cortical bone under compression and 3000 under tension [48]. The trabecular bone’s threshold limits are approximately 7000–8000 µε in both tension and compression because it can tolerate more strain than the cortical bone [49]. Furthermore, the total displacements of prosthetic implants with different materials were examined in this research and compared with a threshold of (100–150 µm [50, 51]) to verify the stability of the fixed restorations.

## Results

### Frontal bite

**Fig. 12:**
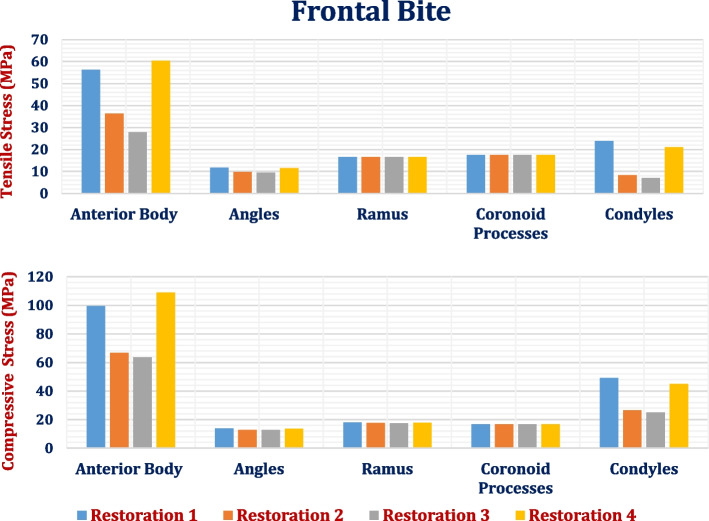
Peak tensile and compressive stresses (MPa) on cortical bone segments in the frontal bite loading scenario

**Fig. 13:**
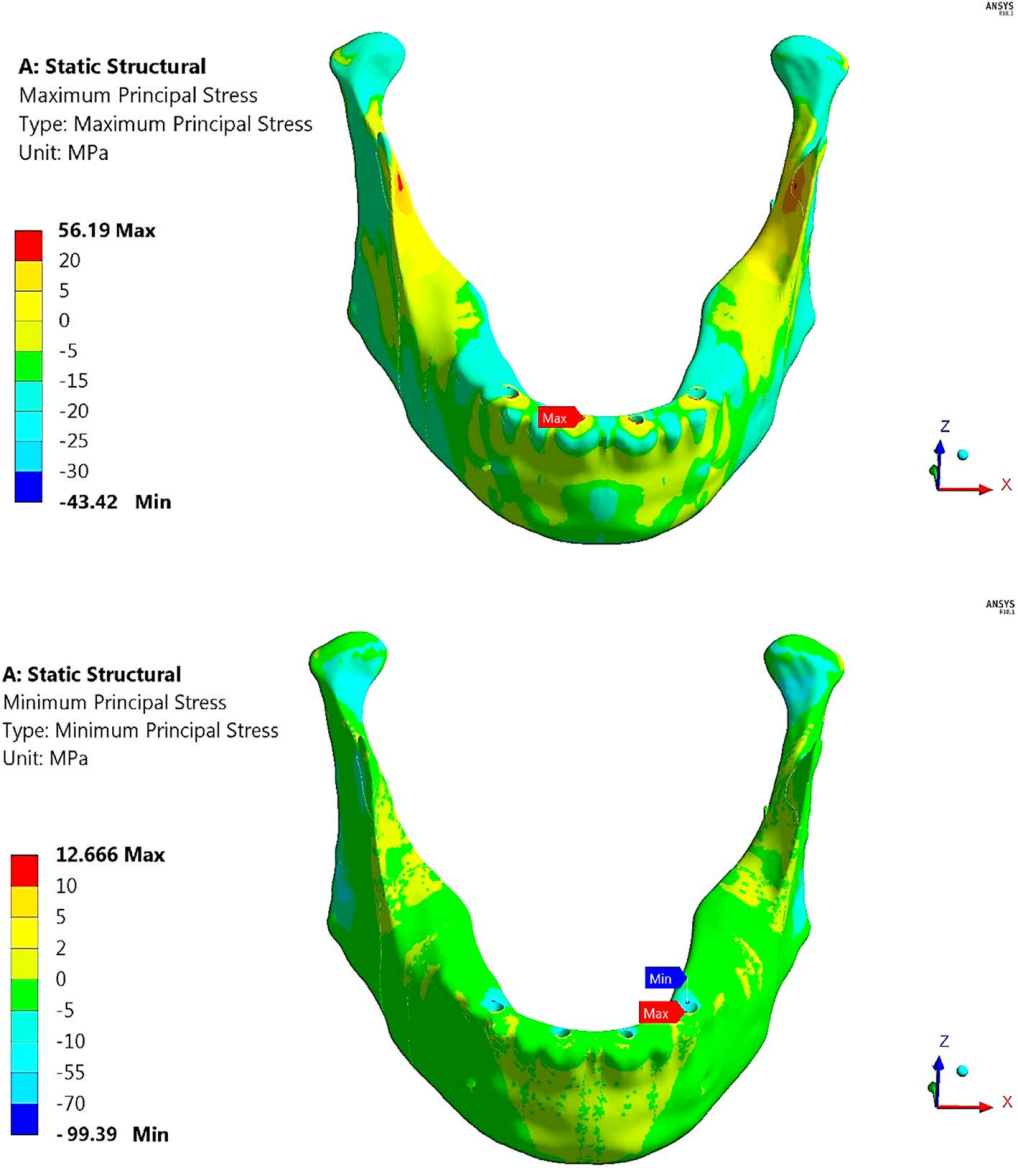
Distributions of maximum and minimum principal stresses on mandible by using restoration.1, under frontal bite loading scenario

As shown in Table 5 and Fig. 12, the anterior body of the cortical bone had the highest stress and strain values compared with the other segments. Moreover, the ramus and coronoid processes had nearly the same tensile and compressive stresses. In addition, the tensile and compressive strains at angles were less than 800 με. Figure 13 illustrates the distributions of maximum and minimum principal stresses on the mandible by using restoration.1, under the frontal bite loading scenario.

**Table 5:** Tensile and compressive stresses (MPa) and strains (με) on cortical bone segments in the frontal bite loading scenario

			**Restoration.1**	**Restoration.2**	**Restoration.3**	**Restoration.4**
**Stress**	**Anterior** **Body**	**Tensile**	56.19	36.36	27.85	60.34
		**Compressive**	99.39	66.71	63.68	108.81
	**Angles**	**Tensile**	11.73	9.75	9.50	11.46
		**Compressive**	13.85	12.80	12.67	13.70
	**Ramus**	**Tensile**	16.63	16.6	16.6	16.64
		**Compressive**	17.93	17.57	17.54	17.86
	**Coronoid Processes**	**Tensile**	17.55	17.55	17.55	17.55
		**Compressive**	16.81	16.67	16.66	16.79
	**Condyles**	**Tensile**	23.86	8.27	7.12	20.97
		**Compressive**	49.19	26.59	25.02	44.98
**Strain**	**Anterior** **Body**	**Tensile**	1987	1585	1509	1669
		**Compressive**	3775	2782	2527	3498
	**Angles**	**Tensile**	667	555	541	651
		**Compressive**	777	715	707	768
	**Ramus**	**Tensile**	1124	1116	1111	1119
		**Compressive**	1131	1121	1120	1130
	**Coronoid Processes**	**Tensile**	1122	1114	1114	1149
		**Compressive**	980	976	976	2238
	**Condyles**	**Tensile**	1176	446	435	1112
		**Compressive**	2437	1416	1329	2400

Restoration.3 (Table 5) decreased the tensile and compressive stresses on cortical bone by (23.4 & 4.5%) on the anterior body, (2.5 & 1.01%) on the angles, and (13.9 & 5.9%) on the condyles compared with titanium restoration.2, whereas the stresses on the ramus and coronoid processes were nearly not changed. As a result, the tensile and compressive strains on cortical bone decreased by (4.75 & 9%) on the anterior body, (2.5 & 1.11%) on the angles and (2.46 & 6.14%) on the condyles.

In contrast, compared with the traditional restoration (restoration.2), restoration.1 increased the tensile and compressive stresses on cortical bone by (54.52 & 48.99%) on the anterior body, (20.3& 8.2%) on the angles, (0.18& 2.04%) on the ramus, and (188 & 84.99%) on the condyles, with nearly no change in the stresses of coronoid processes. As a result, the tensile and compressive strains on the cortical bone changed by (25.36 & 35.68%) on the anterior body, (20.18 & 8.67%) on the angles, (0.7 & 0.8%) on the ramus and (163.67 & 72.10%) on the condyles. Table 5 also illustrates that the restoration.4 increased the stresses and strains on all segments of the cortical bone compared with the titanium restoration.2. Using all restorations for cortical bone, the peak tensile and compressive stresses did not exceed the critical limits of 100 and 140 MPa in tension and compression. In addition, the peak tensile and compressive strains did not exceed the limits of 3000 με and 5000 με in tension and compression, respectively.

**Fig. 14:**
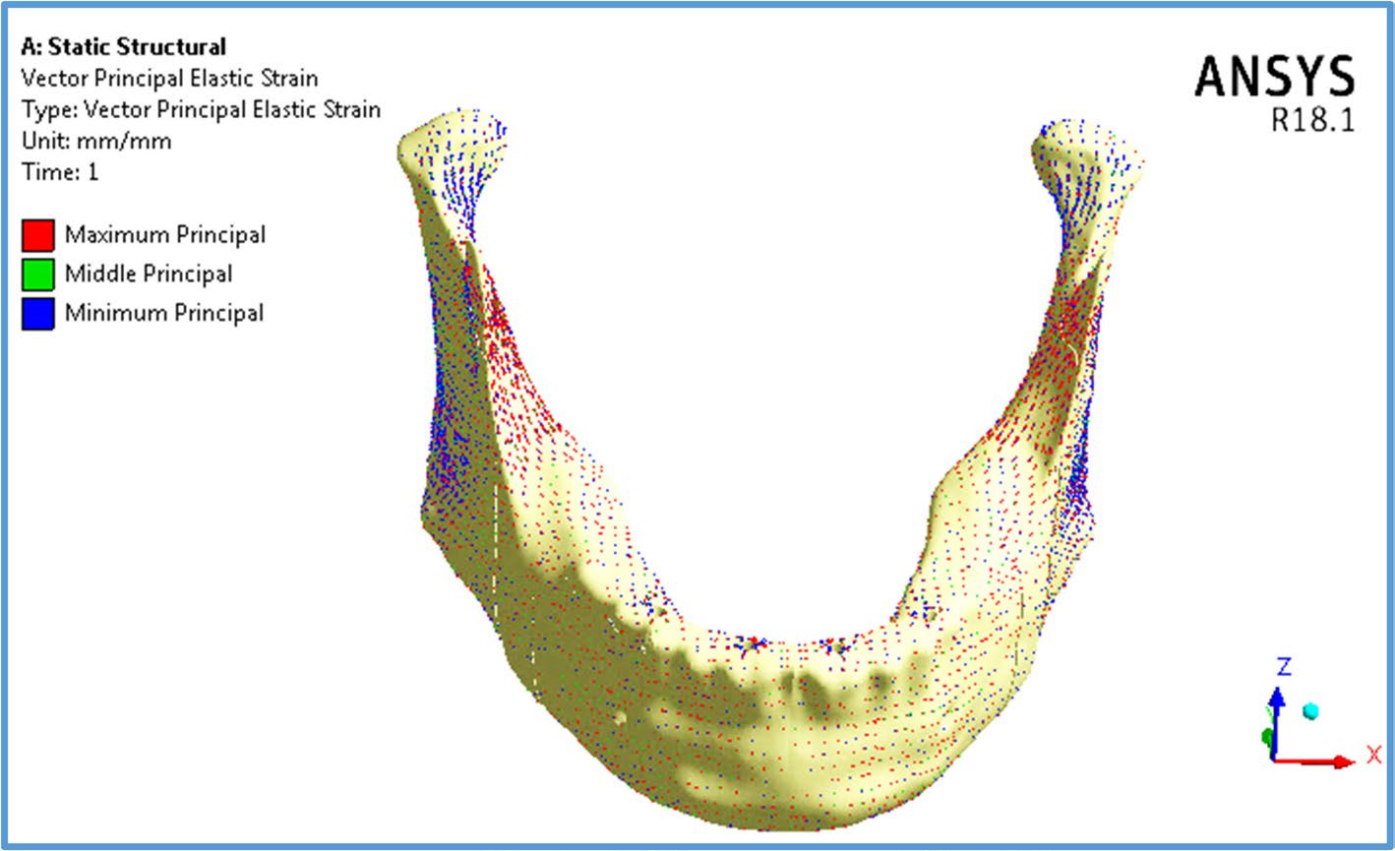
Principal strain vector on the mandible using restoration.1, under the frontal bite loading scenario

Figure 14 illustrates the vector of principal strain on the mandible by using restoration.1, under frontal bite loading scenario. The red and blue arrows indicate tensile and compressive strains, respectively. As shown in Fig. 14, the tensile strains on the anterior body were distributed more than the compressive strains, except around the holes of the implants, where the tensile and compressive strains were approximately evenly distributed. Moreover, compressive strains were more distributed on the exterior sides of the ramus, angles, and coronoid processes, whereas tensile strains were more distributed on the interior sides.

**Fig. 15:**
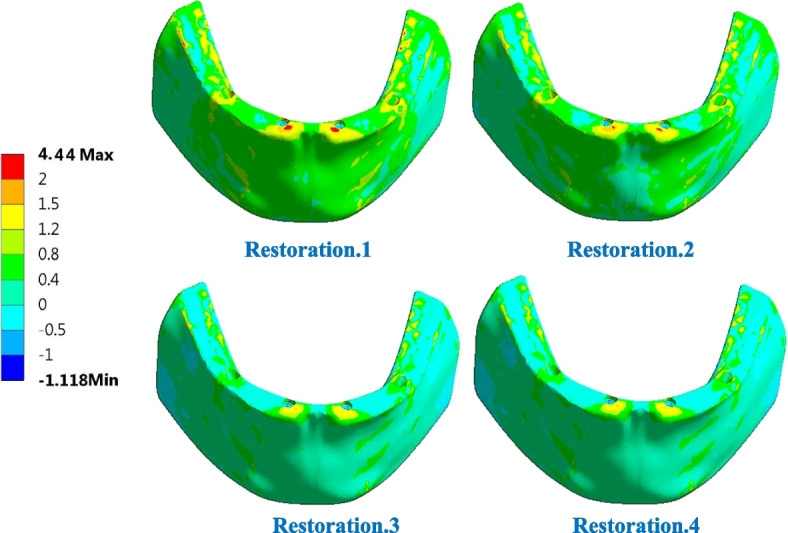
Distribution of maximum principal stress on the trabecular bone using all restorations under frontal bite loading scenario

**Fig. 16:**
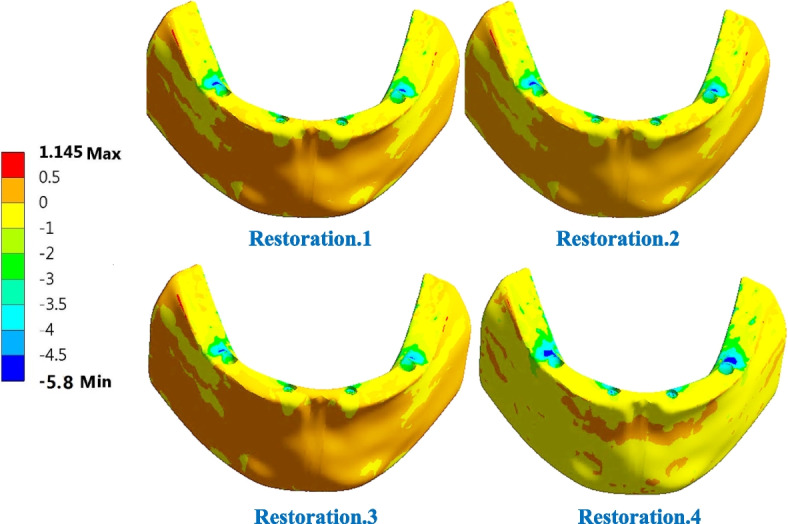
Distribution of minimum principal stress on the trabecular bone using all restorations under frontal bite loading scenario

For trabecular bone (Table 6), the tensile and compressive stresses were changed by (2.06 & 1.91%), (-42.52 & -0.192%) and (-44.13 & 11.32%) using restoration.1, restoration.3, and restoration.4, compared to the traditional restoration.2. Consequently, the tensile and compressive strains were changed by (3.1 & 1.63%), (-13.66& -0.44%) and (-9.52 & 2.22%), respectively. Table 6 also illustrates that the tensile and compressive stresses were within the allowable limits of (12 & 16 MPA in tension and compression) and that the strains did not exceed 700με, which was far from the limits of (7000–8000 με in both tension and compression). Figures 15  and 16 illustrate the distributions of maximum and minimum principal stresses on the trabecular bone using all restorations under a frontal bite loading scenario. As shown in the figures, restoration.1 produced the highest tensile stress on the trabecular bone compared with traditional restoration.2, while restoration.4 produced the highest compressive stress.

**Table 6:** Tensile and compressive stresses (MPa) and strains (με) on trabecular bone, under frontal bite loading scenario

		**Restoration.1**	**Restoration.2**	**Restoration.3**	**Restoration.4**
**Stress**	**Tensile**	4.44	4.35	2.5	2.43
	**Compressive**	5.31	5.21	5.2	5.80
**Strain**	**Tensile**	498	483	417	437
	**Compressive**	686	675	672	690

### Maximum intercuspation

**Fig. 17:**
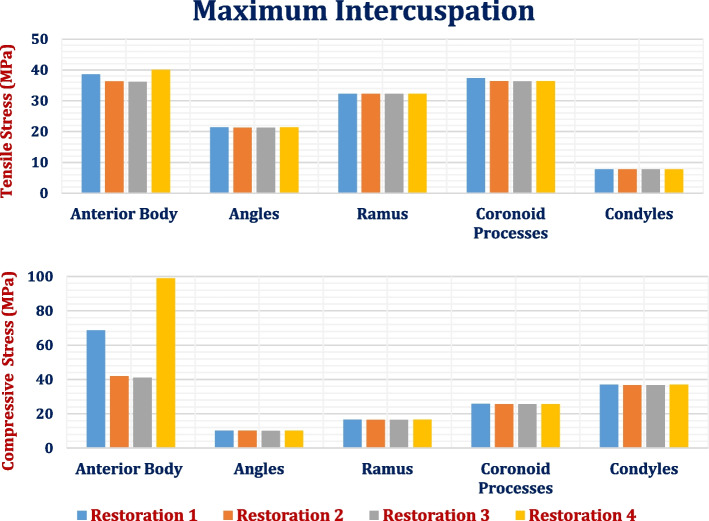
Peak tensile and compressive stresses (MPa) on cortical bone segments in max intercuspation loading scenario

In the second scenario, maximum intercuspation was stimulated as shown in Tables 7 & 8 and Fig. 17, where 450 N vertical force was distributed on the right and left posterior teeth to stimulate bilateral mastication. As shown in tables, the highest compressive stresses and strains were observed in the anterior body of the cortical bone. In contrast, the lowest tensile stresses and strains were observed in the condyles when all restorations were used.

**Table 7:** Tensile and compressive stresses (MPa) and strains (με) on cortical bone segments under max intercuspation loading scenario

			**Restoration.1**	**Restoration.2**	**Restoration.3**	**Restoration.4**
**Stress**	**Anterior** **Body**	**Tensile**	38.56	36.27	36.15	40.02
		**Compressive**	68.63	41.93	41	98.87
	**Angles**	**Tensile**	21.35	21.3	21.3	21.33
		**Compressive**	10.13	10.09	10.08	10.13
	**Ramus**	**Tensile**	32.22	32.20	32.20	32.26
		**Compressive**	16.54	16.50	16.50	16.59
	**Coronoid Processes**	**Tensile**	37.30	36.34	36.25	36.34
		**Compressive**	25.80	25.65	25.65	25.67
	**Condyles**	**Tensile**	7.72	7.70	7.70	7.73
		**Compressive**	37	36.80	36.78	37.01
**Strain**	**Anterior** **Body**	**Tensile**	2907	1789	1788	2948
		**Compressive**	2490	2370	2366	4568
	**Angles**	**Tensile**	1128	1128	1127	1128
		**Compressive**	663	662	661	663
	**Ramus**	**Tensile**	1693	1692	1692	1695
		**Compressive**	882	880	880	885
	**Coronoid Processes**	**Tensile**	2198	2100	2095	2101
		**Compressive**	1507	1504	1504	1505
	**Condyles**	**Tensile**	768	762	760	767
		**Compressive**	1871	1860	1859	1871

By using restoration.3, the tensile and compressive stresses and strains were almost not changed, compared to the titanium restoration.2. In contrast, using the restoration.1, the tensile and compressive stresses on the anterior body of the cortical bone increased by (6.3& 63.67%), and thus the tensile and compressive strains increased by (62.49& 5.06%), while the other segments slightly changed (less than 5%), compared to the titanium restoration.2. Restoration.4 significantly increased the tensile and compressive stresses and strains on the anterior body of the cortical bone by (10.339 & 135.79%) and (64.78 & 92.74%) respectively. In addition, the values of tensile and compressive strains on the cortical bone were near the critical limits of (3000 & 5000με) which may induce microdamage in the anterior body. For the ramus, condyles, and angles, the tensile and compressive strains were less than 2000με by using all restorations.

**Fig. 18:**
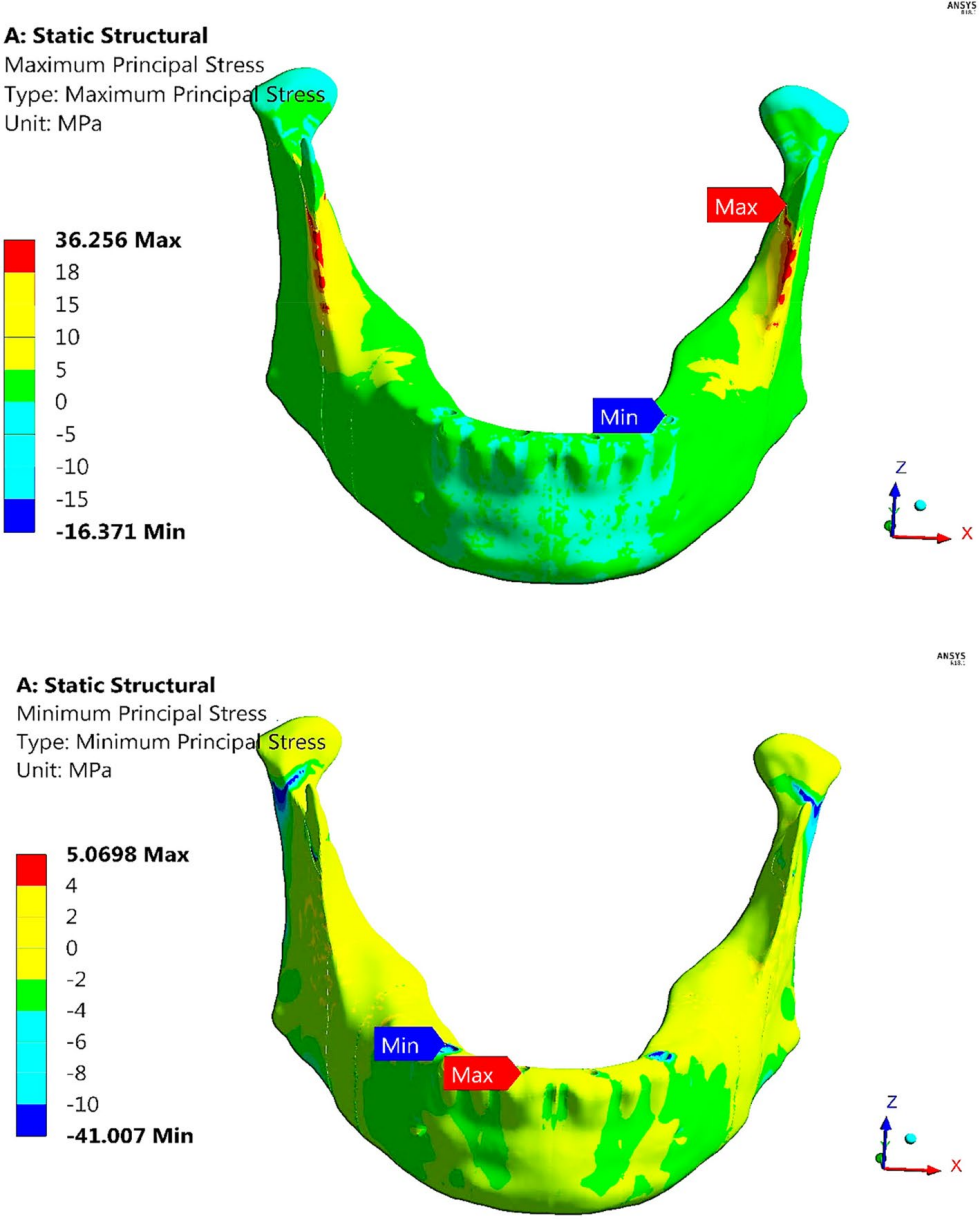
Distributions of maximum and minimum principal stresses on the mandible using restoration.3, under the maximum intercuspation loading scenario

**Fig. 19:**
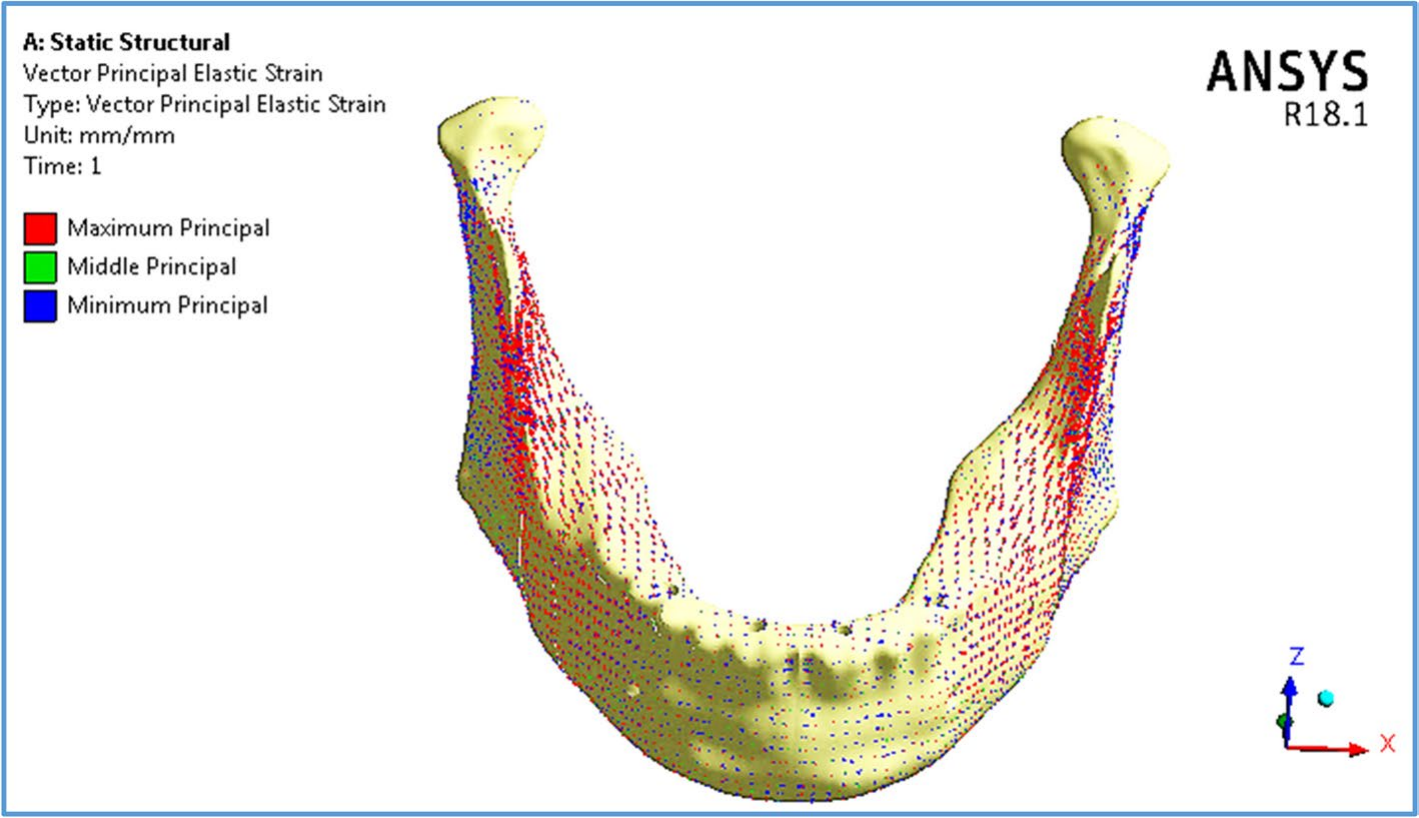
Principal strain vector on the mandible using restoration.3, under the maximum intercuspation loading scenario

Figure 18 illustrates the distributions of maximum and minimum principal stresses on the mandible using restoration.3, under the maximum intercuspation loading scenario. The figure illustrates that the peak tensile stress was observed at the coronoid process, and the peak compressive stress was observed at the anterior body. Figure 19 illustrates the principal strain vector on the mandible via restoration.3. As shown in Fig. 19, the tensile strains were more distributed around the coronoid processes than the compressive strains. In addition, for the other segments, the tensile and compressive strains were approximately evenly distributed.

For the trabecular bone (Table 8), the tensile and compressive stresses and strains were increased by (1.46& 2.133%) and (1.3& 2.09%) respectively, using restoration.1. In contrast, restoration.3 slightly decreased the stresses and strains on the trabecular bone, while restoration.4 changed the tensile and compressive stresses and strains by (-2.2& 1.42%) and (1.2& 1.99%) respectively. However, using all restorations, the tensile and compressive stresses and strains on the trabecular bone were within the allowable limits.

**Table 8:** Tensile and compressive stresses (MPa) and strains (με) on trabecular bone under max intercuspation loading scenario

		**Restoration.1**	**Restoration.2**	**Restoration.3**	**Restoration.4**
**Stress**	**Tensile**	4.15	4.09	4.01	4.00
	**Compressive**	4.31	4.22	4.20	4.28
**Strain**	**Tensile**	1007	994	993	1006
	**Compressive**	975	955	954	974

### Chin impact

**Fig. 20:**
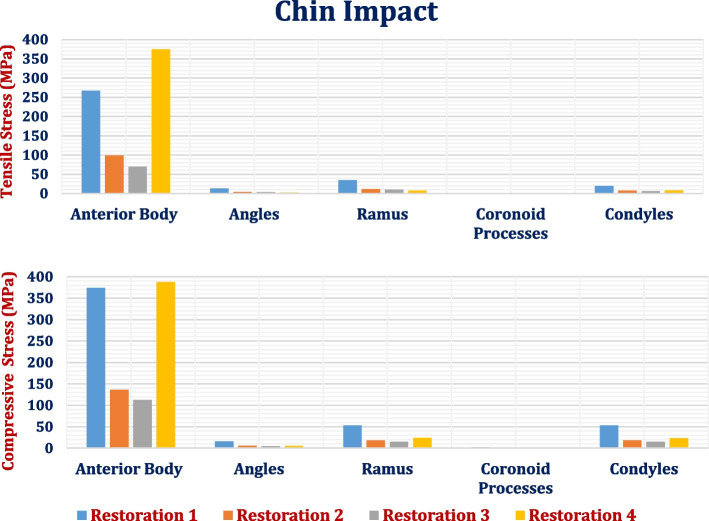
Peak tensile and compressive stresses (MPa) on cortical bone segments in chin impact loading scenario

The values of the tensile and compressive stresses and strains on the cortical and trabecular bones under the chin impact force are illustrated in Tables 9 and 10 and Fig. 20, using four different restorations. In this impact case, the anterior body of the cortical bone had the highest tensile and compressive stresses and strains. By using titanium restoration.2, the tensile and compressive stresses were (98.63 & 136.53 MPa). By using restoration.1 and restoration.4, respectively, these tensile and compressive stresses were greatly increased to (266.76 & 374.25 MPa) and (374.57 & 387.3 MPa), which exceeded the critical limits and caused destruction in the anterior body of the cortical bone.

**Table 9:** Tensile and compressive stresses (MPa) and strains (με) on cortical bone segments during the chin impact scenario

			**Restoration.1**	**Restoration.2**	**Restoration.3**	**Restoration.4**
**Stress**	**Anterior** **Body**	**Tensile**	266.76	98.63	69.79	374.57
		**Compressive**	374.25	136.53	112.38	387.3
	**Angles**	**Tensile**	13.08	4.17	3.31	2.12
		**Compressive**	15.89	5.92	4.91	5.13
	**Ramus**	**Tensile**	34.48	11.45	9.80	7.38
		**Compressive**	53.19	17.84	15.18	23.65
	**Coronoid Processes**	**Tensile**	1.71	0.80	0.65	0.71
		**Compressive**	1.99	0.70	0.57	0.49
	**Condyles**	**Tensile**	19.79	7.6	6.26	8.05
		**Compressive**	53.13	17.93	15.14	23.38
**Strain**	**Anterior** **Body**	**Tensile**	4737	2450	2115	5231
		**Compressive**	8958	2824	2582	9289
	**Angles**	**Tensile**	596	190	154	163
		**Compressive**	924	343	285	290
	**Ramus**	**Tensile**	1468	499	424	529
		**Compressive**	2545	895	754	1140
	**Coronoid Processes**	**Tensile**	1005	452	376	445
		**Compressive**	1024	381	311	279
	**Condyles**	**Tensile**	1543	521	440	656
		**Compressive**	3231	1081	915	1403

**Table 10:** Tensile and compressive stresses (MPa) and strains (με) on trabecular bone during the chin impact scenario

		**Restoration.1**	**Restoration.2**	**Restoration.3**	**Restoration.4**
**Stress**	**Tensile**	10.3	9.3	8.6	10.9
	**Compressive**	21.7	15.9	10.8	26.1
**Strain**	**Tensile**	4959	4300	4279	5251
	**Compressive**	6800	6241	5802	7457

Although titanium restoration.1 greatly increased the tensile and compressive stresses and strains on the angles, ramus, coronoid processes, and condyles compared to titanium restoration.2, these values were within the allowable limits. In addition, restoration.4 increased the tensile and compressive stresses and strains on most cortical bone segments, but these values were within the allowable limits. For cortical bone, restoration.3 decreased the tensile and compressive stresses by (29.24 & 17.68%), (20.62 & 17%), (14.4 & 14.9%), (18.75 & 18.57%), and (17.63 & 15.56%) on the anterior body, angles, ramus, coronoid processes, and condyles, respectively. Consequently, the tensile and compressive strains decreased by (13.67 & 8.56%), (18.94 & 16.91%), (15.03& 15.75%), (16.8 & 18.37%) and (15.54 & 15.35%) on the segments of cortical bone. For the anterior body, the peak tensile and compressive strains exceeded the limits of 3000 με and 5000 με in tension and compression, respectively.

**Fig. 21:**
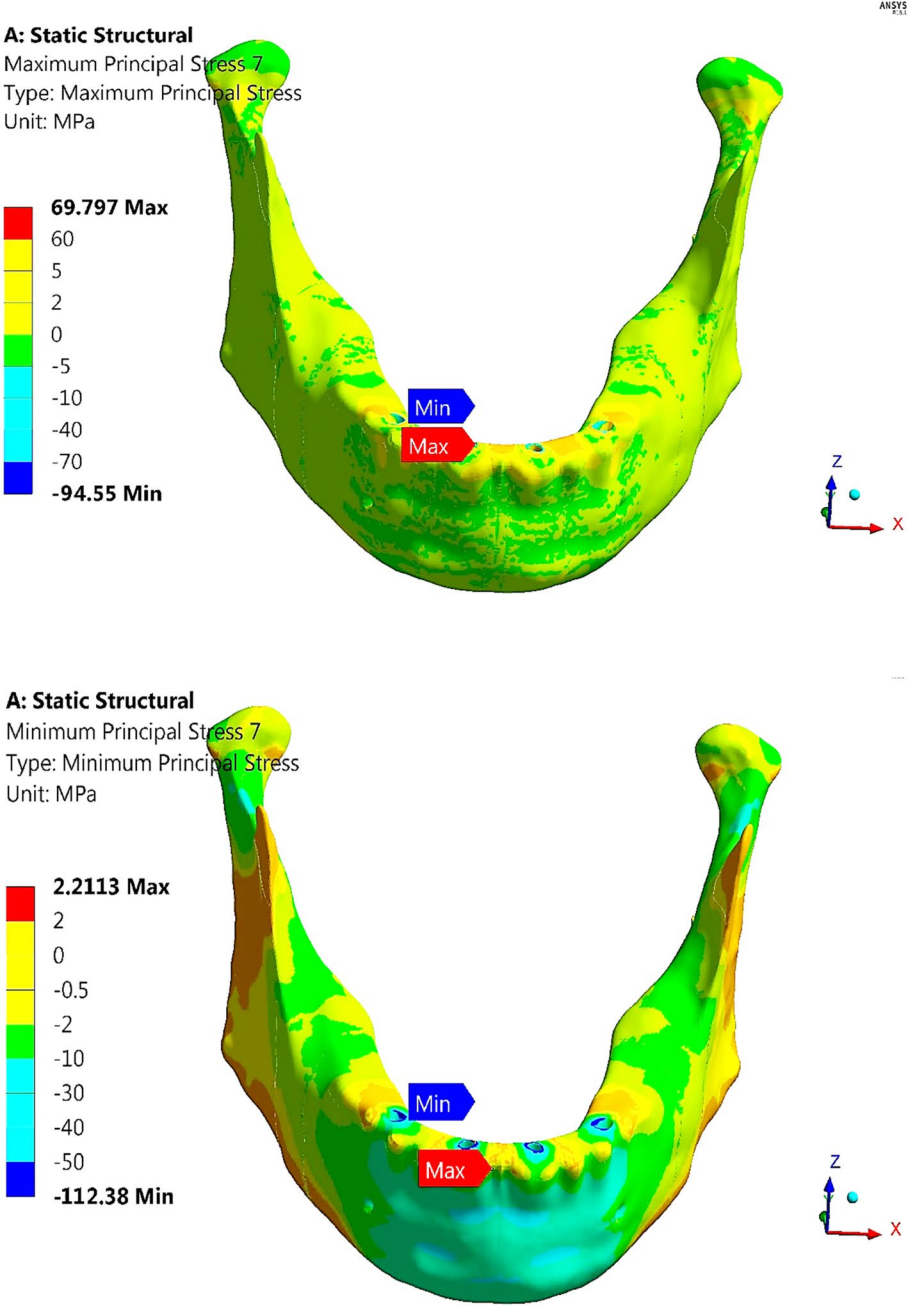
Distributions of maximum and minimum principal stresses on mandible by using restoration.3, under chin impact scenario

**Fig. 22:**
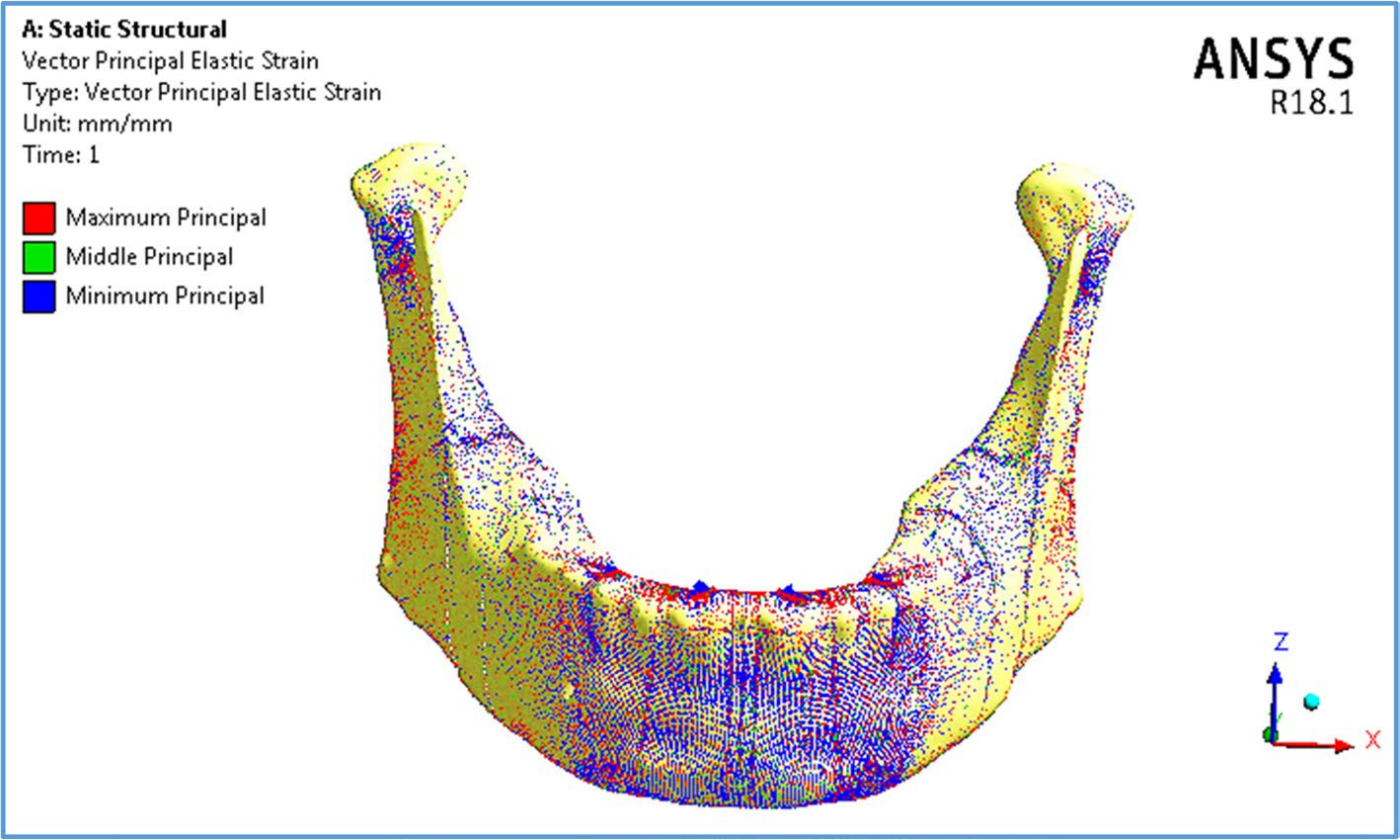
Principal strain vector on the mandible using restoration.3, under the chin impact scenario

Figure 21 illustrates the distributions of the maximum and minimum principal stresses on the mandible using restoration.3, under the chin impact loading scenario. Figure 22 presents the principal strain vector on the mandible via restoration.3. This figure illustrates that the anterior body of the mandible had more tensile strain than the other segments.

**Fig. 23:**
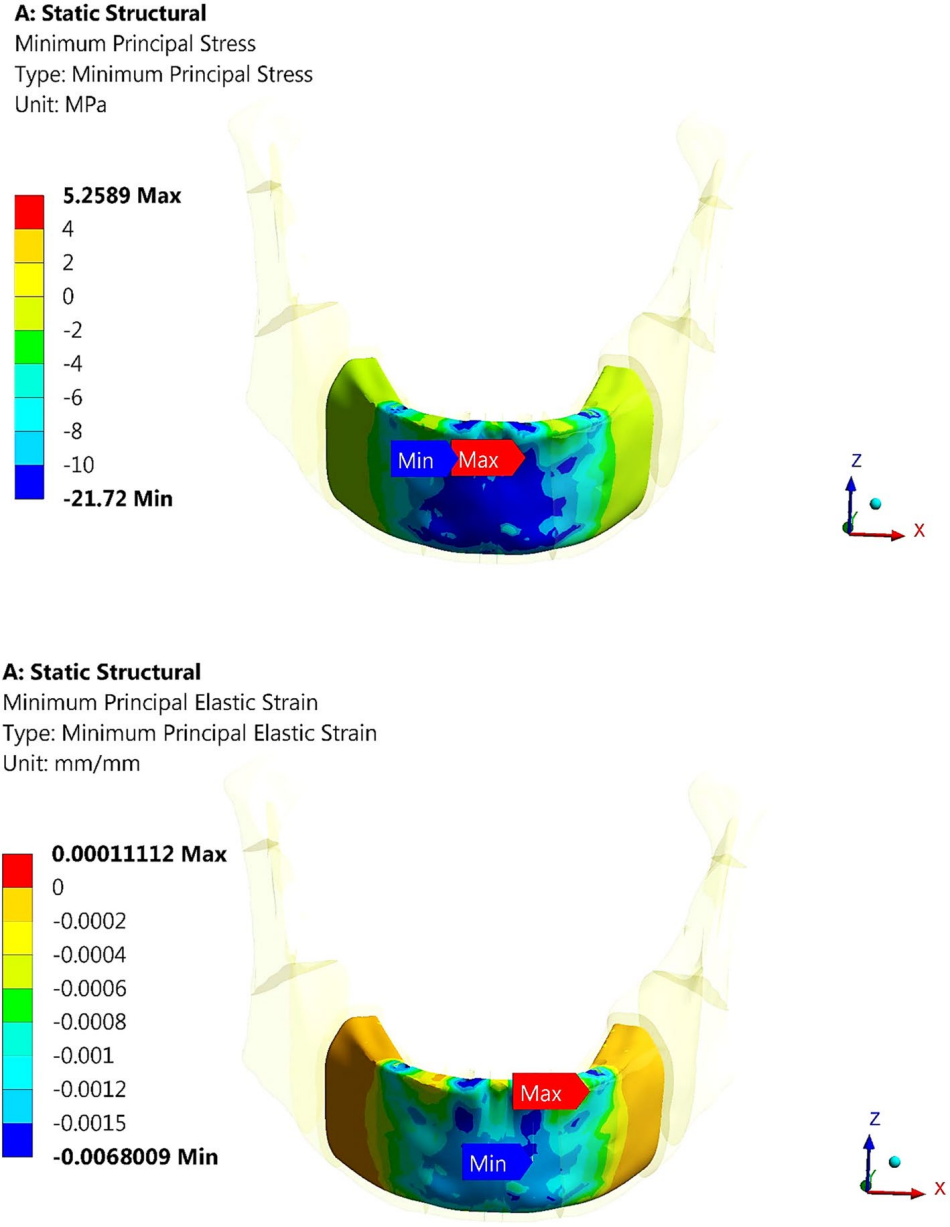
Distribution of minimum principal stress and strain on trabecular bone using restoration.1, under the chin impact scenario

For the trabecular bone (Table 10), the tensile and compressive stresses and strains were increased by (11.17 & 36.26%) and (15.32 & 8.95%), respectively, using the restoration.1. In addition, the tensile and compressive stresses and strains increased by (17.6 & 63.455%) and (22.13 & 19.47%), by using the restoration.4. As shown in Table 10, the values of compressive stresses and strains determined using restoration1 & restoration.4 exceeded the critical limits of 16 & 7000 με which may increase the potential for trabecular bone destruction. In contrast, the restoration.3 decreased the tensile and compressive stresses and strains on the trabecular bone by 6.8,32.32, 0.48 and 7.03%, respectively, compared with the titanium restoration.2. Figure 23 illustrates the distribution of minimum principal stress and strain on trabecular bone by using restoration.1, under chin impact loading scenario.

### Total deformation of the mandible

**Fig. 24:**
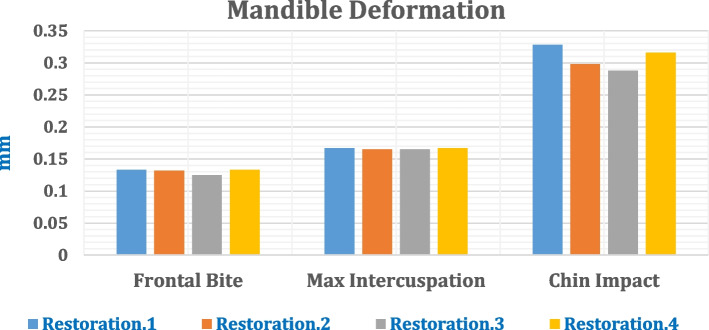
Total maximum deformation of the mandible after four restorations

**Fig. 25:**
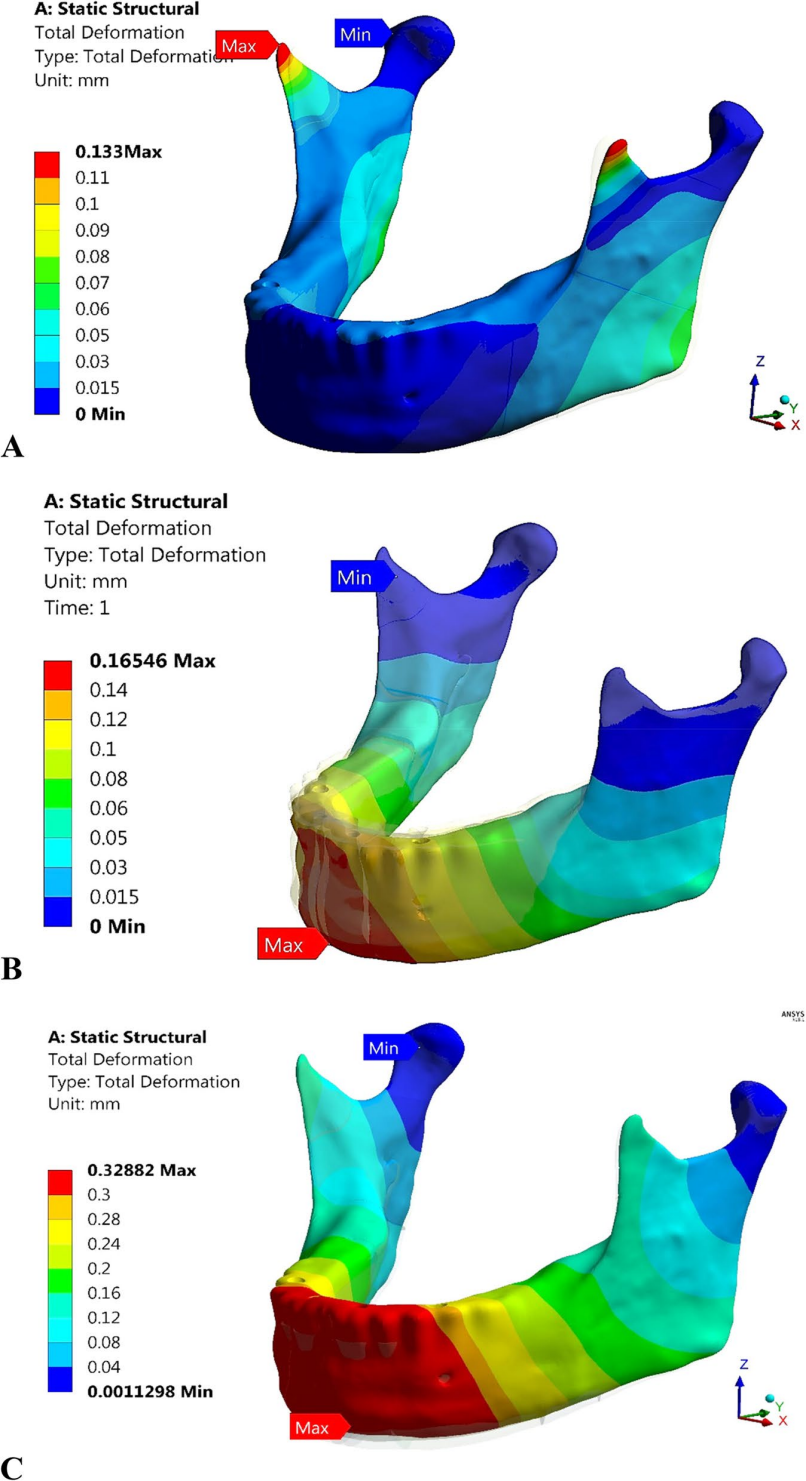
Total deformation of the mandible using restoration.1, under **A**) Frontal bite, **B**) maximum intercuspation, and **C**) Chinese impact scenarios

The maximum deformations of the mandible rehabilitated with four different restorations were calculated (Fig. 24) under the three loading scenarios to determine the changes in the mandibular shape or size due to the applied loads. As shown in Fig. 24, under the frontal bite scenario or the max intercuspation scenario, the total deformations did not exceed 0.17 mm when all restorations were used. Under the chin impact scenario, the total deformation was 0.298 mm using titanium restoration.2. This value increased by 10% and 6% using restorations 1 and 4; however, it decreased by 3.35% by using restoration.3. Figure 25 illustrates the total deformation of mandible by using restoration.1, under all loading scenarios. In the frontal bite loading scenario, the coronoid process had the highest deformation, whereas the condyle had the lowest deformation. In max intercuspation and chin impact loading scenarios, the anterior body had the highest deformation value, whereas the coronoid process (or condyle) had the lowest deformation value.

### Stability of prosthetic restorations

**Fig. 26:**
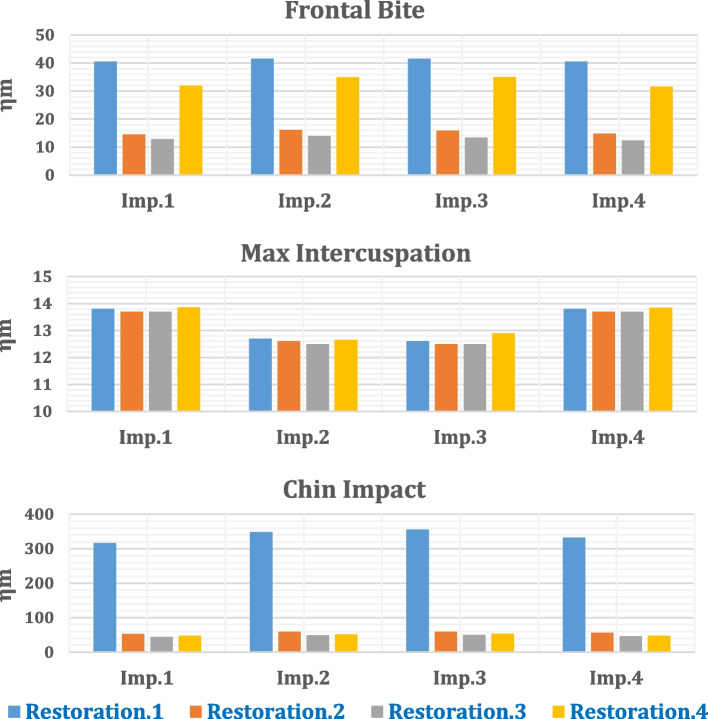
Maximum displacements (μm) of the four implants

Finally, to assess the primary stability of the fixed prosthetic restorations, the maximum values of total displacements (μm) of the four implants were extracted and compared with the critical limits of (100–150 μm), under the three loading scenarios, as shown in Fig. 26. Imp.1&Imp.4 were the posterior implants and Imp.2&Imp.3 were the anterior implants.

In frontal bite and chin impact scenarios when all restorations were used, the anterior implants (Imp.2 & Imp.3) exhibited higher displacements than the posterior implants. In contrast, the posterior implants (Imp.1& Imp.4) exhibited higher displacements than the anterior implants in maximum intercuspation. In addition, zirconia implants exhibited displacements greater than 150 µm in the chin impact loading scenario. This may decrease the stability of the prosthetic restoration.1 and raise the risk of falling out. In contrast, in comparison with titanium implants, the displacements of BIOHPP implants in restoration.3 decreased, increasing the restoration stability.

## Discussion

In the current research, finite element analysis was conducted to evaluate the biomechanical behavior of mandibles that were rehabilitated with four fixed prosthetic restorations made from different materials. These restorations were restoration.1 (zirconia frameworks and implants with acrylic teeth), restoration.2 (titanium frameworks and implants with acrylic teeth), restoration.3 (BIOHPP frameworks and implants with acrylic teeth), and restoration.4 (zirconia framework and BIOHPP implants with zirconia teeth). The responses of the mandibles were assessed under both normal and impact loading conditions.

Mandibular fractures are the most common facial fractures, with a high prevalence of condyle fractures [3, 52]. Nevertheless, the fracture site may also be in the coronoid process, angle, body, or midline of the mandible. Mandibular fractures are common in older patients, with an incidence ranging from 27.8% to 43.3%. In addition, males are more affected than females (60% vs. 39.4%) [53–56]. Therefore, each loading scenario necessitates evaluating the mandibular biomechanical behavior to avoid fractures.

Hedesiu et al. [32] conducted a finite element analysis using computer-aided design (CAD) models to examine the biomechanical behavior of the mandible under standardized trauma conditions and assess the biomechanical response of the normal, partial, and total edentulous mandible. The findings showed that all stress levels in the lateral impact scenario were higher than the critical value, which indicates the point at which the mandible’s condyle region is vulnerable to fracture. In addition, unilateral or bilateral mandibular edentation had relatively similar stress levels for the lateral impact.

For edentulous patients, to restore quality of life, “complete dentures”, “implant removable dentures” and “implant-supported fixed restorations” are used as acceptable treatment options [5, 9, 10]. In fixed prosthetic restorations, the longevity, stability, and success of the restoration are significantly influenced by the material selection [13]. Titanium’s superior biocompatibility, corrosion resistance, and stiffness make it the preferred material for most prosthetic parts, such as screws, implant systems, and frames. Ceramics and polymers have recently been employed in fixed restorations as alternatives to titanium [18, 19].

Zirconia can be used in the fabrication of posts, crowns, abutments, frameworks, brackets, artificial teeth, and implants as an alternative to titanium. Compared with metal posts, zirconia posts minimize pain and the possibility of inflammation by preventing corrosive reactions in the patient’s mouth and any surrounding tissue. For crowns, abutments, and prosthetic teeth, zirconia is the material of choice due to its strength, durability, and esthetic appearance. In addition, the ceramic brackets act as restraints against the teeth and suspenders wires. Additionally, because of its exceptional chemical stability and biocompatibility, zirconia is a suitable material for use in dental implants. [20, 22]

In the fabrication of frameworks, according to [57], the zirconia framework showed biomechanical behavior similar to that of the titanium framework. In contrast, the zirconia framework in Kelkar et al.’s study [58] produced the lowest stress values under axial and oblique forces compared to the titanium framework. The disadvantages of zirconia include its high modulus of elasticity, high density, high cost, and long production time.

Recently, polymeric materials have played a major role in most areas of restorative dentistry, including the construction of removable dentures and fixed prostheses [24,25.27,28]. These materials have good mechanical, chemical, thermal, and electrical capabilities, as well as good esthetics. They are also radiolucent and biocompatible both in vivo and in vitro. Polymethyl methacrylate (PMMA) is commonly used for prosthetic dental applications, such as the fabrication of artificial teeth, denture bases, dentures, obturators, and orthodontic retainers. Furthermore, it can be prepared in an ambient setting (such as a dental office or operating theater). However, PMMA is a brittle polymer, and it does not seem appropriate for clinical use [14].

New high-performance polymeric materials, PEEK (poly ether ether ketone) and PEKK (poly ether ketone ketone), play a significant role in most restorative dental applications [59, 60]. These materials are anticipated to address stress-related issues in bone because of their low modulus of elasticity and ability to absorb shock. In Villefort et al. [59] work, the behavior of polyetherketoneketone (PEKK) and polyetheretherketone (PEEK) prosthetic frameworks was investigated using finite element analysis. A three-dimensional maxillary model with four implants supported by a polymeric bar was simulated under a 500 N force applied to the first left molar. The microstrain and von Mises stresses were selected as the analysis criteria. The results showed that the superior shock absorbance of PEKK resulted in a lower stress concentration on the prosthetic screw and base, thereby reducing the fracture risk of the acrylic base and screw loosening. In contrast, a lower stress concentration was observed in the PEEK frameworks.

Shash et al. [61], used the finite element method to perform stress–strain analysis on a mandible rehabilitated with a hybrid prosthesis, utilizing PEEK material rather than titanium to fabricate the “All on four” parts, using various densities of cancellous bone. To stimulate the different mastication mechanisms, a 300 N vertical force was applied unilaterally, bilaterally, and anteriorly. The results showed that compared with titanium, PEEK enhanced mucosal stress and decreased bone tissue stress and strain. Consequently, this material has recommended for the fabrication of “All on four” parts, especially in the low-density model.

In Heboyan et al*.* [62] study, the stress–strain pattern of zygomatic dental implants supporting different superstructures were assessed and compared using 3D finite element analysis (FEA), under 500 N axial load. A 3D model of the edentulous maxilla with four dental implants supported by a U-shaped bar was constructed using computer-aided design (CAD) software. Different materials were simulated for the superstructure which were cobalt-chrome (CoCr) alloy, titanium alloy (Ti), zirconia (Zr), carbon-fiber polymers (CF), and polyetheretherketone (PEEK). The results demonstrated that all superstructure materials resulted in homogeneous strain and could reconstruct the edentulous maxilla. Besides, stiffer materials, such as Zr, CoCr, and Ti, can reduce the stress in zygomatic implants and prosthetic screws.

Tribst et al. [63]*.* evaluated the effect of framework material and distal implant angulation on the stress concentration of an All-on-4 full-arch prosthesis using the finite element method. The framework was stimulated with different materials (Cobalt-chrome, Yttria-stabilized tetragonal zirconia polycrystal [Y-TZP], and polyetheretherketone [PEEK]). A vertical load of 200 N was applied in the distal region of the cantilever arm, and stress was evaluated in the Von Misses for prosthesis components and the maximum and minimum principal stresses for the bone. The results demonstrated that YTZP and CoCr concentrated stress in the framework structure and reduced the stress in the prosthetic screw compared to PEEK.

In addition, Mourad et al*.* [64] conducted a study to evaluate the clinical changes in peri-implant soft tissue during the first year after occlusal loading and the ridge base relationship after 3 years for a mandibular hybrid prosthesis with PEEK. The results demonstrated that the full-arch PEEK framework of the hybrid prosthesis, which utilizes the All-on-Four concept, is an acceptable treatment approach.

PEEK properties can be rehabilitated to suit biological demands by adding other materials such as glass fibers (GFR-PEEK), carbon fibers (CFR-PEEK), or ceramic fillers (BIOHPP) [24, 25]. BIOHPP is currently one of the safest and most scientifically approved materials for use in both fixed and removable restorations because of its superior mechanical properties and biocompatibility. Furthermore, BIOHPP is distinguished by its lightweight, low cost, capacity to withstand shock, ease of maintenance, and compatibility with imaging methods [26–28]. According to [38], BIOHPP can substitute titanium in the construction of hybrid prosthesis frameworks due to its ability to attenuate stresses transmitted to infrastructures and bone tissues. However, further studies are required to investigate the possibility of using this soft material as an alternative to metals and ceramics in dental and orthopedic surgery.

In the current research, the mandible was divided into five segments which were the anterior body (including the inner volume of the trabecular bone), ramus, angle, coronoid process, and condyle, and each segment was stimulated with orthotropic properties. In mesh generation, the division of the mandible into a minimum of 30,000 finite elements was necessary to obtain a high-precision analysis, as demonstrated by Choi et al*.* [65]. In contrast, 30,119 elementary tetrahedral states were reported by Torreira et al*.* [66]. In the current research, mandible meshing with 380,637 elements and 522,057 nodes was created using an adaptive function with element sizes ranging from 0.4 to 2 mm.

The mandible responses were assessed under three different loading scenarios. The first was the frontal bite, in which six masticatory muscles (temporalis, masseter, and pterygoid) were activated, and a stiff body was pressed by the six frontal artificial teeth. The second loading scenario was the normal maximum intercuspation by posterior premolar and molar teeth. The third loading scenario involved the impact force applied to the chin area due to a frontal strike or fall. From the extraction of the tensile and compressive stresses and strains, as well as the total deformations, the biomechanical behavior and clinical situations were studied and described for all mandibular segments under the three loading scenarios. To assess the stability of fixed restorations under various loading scenarios, the total displacements of implants made of various materials were computed.

The results of this study demonstrated that in the frontal bite loading scenario, the anterior body of the cortical bone exhibited the highest stress and strain values compared with the other segments. Furthermore, the tensile and compressive stresses in the ramus and coronoid process parts were almost not changed. Furthermore, the tensile and compressive strains at the angles were less than 800με. The maximum tensile and compressive stresses and strains achieved by employing all restorations were not above the critical limits of cortical bone, which are ((100 MPa & 3000 με) in tension and (140 MPa & 5000 με) in compression). For trabecular bone, the tensile and compressive stresses were within the allowable limits of (12 & 16 MPA in tension and compression), and the strains did not exceed 700με, which was far from the limits of (7000–8000 με in both tension and compression).

In maximum intercuspation, when all restorations are used, the condyles exhibited the lowest tensile stress and strain. By employing restoration.4(ZR-BIOHPP) and restoration.1(zirconia), the values of tensile and compressive strains on the cortical bone were close to the critical limits of (3000&5000 με), which may cause micro damage in the anterior body. Using all restorations, the tensile and compressive strains on the ramus, condyles, and angles were less than 2000 με. In addition, for trabecular bone, the values of the tensile and compressive stresses and strains on the bone were within the allowable limits when using all restorations.

**Fig. 27:**
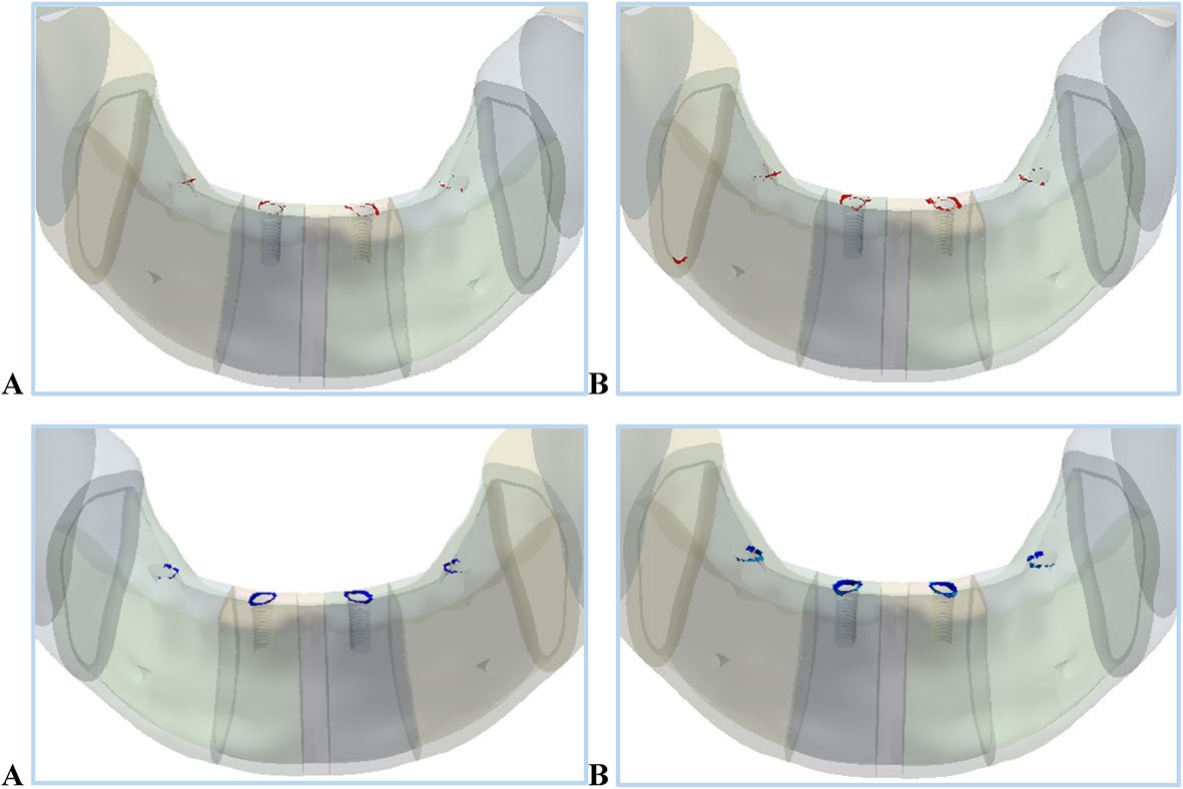
Expected fractured areas in the anterior body by tension(red) and compression (blue) using: (**A**) restoration.1 & (**B**) restoration.4, under a chin impact force

In the chin impact loading scenario, the anterior body of the cortical bone had the highest tensile and compressive stresses and strains and exceeded the critical limits, causing bone failure when using restorations 1 and 4. Figure 27 presents the expected fracture areas under tension(red) and compression (blue). Fracture was expected to occur in weak areas in the anterior body (the holes drilled for the implants), as shown in Fig. 27. Although the restoration.1 greatly increased the values of tensile and compressive stresses and strains on the angles, ramus, coronoid processes, and condyles, compared to titanium restoration.2, these values were within the allowable limits. For trabecular bone, the compressive stresses and strains measured using restorations 1 and 4 exceeded the critical limits, which may increase the potential for trabecular bone destruction.

This study had some limitations related to restoration, as changing the type, number, or location of implants may change the results. In addition, the study was conducted under static loading. However, materials may exhibit distinct behaviors under the cyclic loading like that occurs during mastication movements. Hence, future research is required to examine different combinations of external forces at different bone densities and types of restorations.

For all loading scenarios, restoration.3 (BIOHPP restoration) decreased the tensile and compressive stresses and strains on cortical and trabecular bone compared to traditional titanium restoration.2 Conversely, restoration.1(zirconia restoration) increased the stresses and strains on all segments. In addition, Zirconia implants exhibited displacements of more than 150 µm in the chin impact scenario, decreasing the stability of prosthetic restoration.1 and increasing the risk of falling out.

## Conclusion

Within the limitations of this research, the following were concluded:

1. The anterior body of the cortical bone exhibited the highest stress and strain values compared with the other segments.
2. In the frontal bite loading scenario, the tensile and compressive stresses in the ramus and coronoid process were nearly equal. In addition, the maximum tensile and compressive stresses and strains on the cortical and trabecular bones were within the allowable limits by employing all restorations.
3. In maximum intercuspation, when all restorations were used, the condyles exhibited the lowest tensile stress and strain. For the anterior body of the mandible, the tensile and compressive strains on the cortical bone were close to the critical limits when using restoartions1&4. The stresses and strains in other segments and trabecular bone were within the allowable limits.
4. In the chin impact loading scenario, the anterior body of the cortical and trabecular bones exhibited high tensile and compressive stresses and strains, causing bone failure, using restorations1&4
5. Compared to the traditional restoration.2, restoration.3 (BIOHPP restoration) decreased the tensile and compressive stresses and strains on cortical and trabecular bone, unlike restoration.1(zirconia restoration) and restoration.4(ZR-BIOHPP).
6. Zirconia implants exhibited displacements of more than 150 µm under the chin impact scenario.
7. BIOHPP is preferred to be used in the fabrication of fixed restorations as an alternative to titanium, especially under impact forces, because it decreases the stresses and strains on the mandible and prevents bone failure.

## Data Availability

The data supporting this study’s findings are available from the corresponding author upon reasonable request.
